# Quercetin Protects Against Transmissible Gastroenteritis Virus-Induced Intestinal Inflammation by Modulating Mitophagy-Driven Mitochondrial Dysfunction

**DOI:** 10.7150/ijbs.116855

**Published:** 2025-10-10

**Authors:** Kang Wang, Zhentao He, Yan Li, Bing Yu, Yuheng Luo, Xiangbing Mao, Hui Yan, Aimin Wu, Junqiu Luo, Jun He

**Affiliations:** 1 Institute of Animal Nutrition, Sichuan Agricultural University, Sichuan Province, Chengdu, 611130, People's Republic of China.; 2 Key Laboratory of Animal Disease-resistant Nutrition, Sichuan Province, Chengdu, 611130, People's Republic of China.

**Keywords:** Inflammation, Intestinal epithelial cells, Mitochondrial dysfunction, Mitophagy, Quercetin, Transmissible gastroenteritis virus

## Abstract

Transmissible gastroenteritis virus (TGEV), an enteropathogenic α-coronavirus, causes severe disruption of the intestinal epithelium and diarrhea in neonatal piglets. Despite growing evidence linking mitochondrial dysfunction to coronavirus-induced inflammation, the role of mitophagy-mediated mitochondrial regulation in TGEV pathogenesis remains largely unclear. Here, we conducted a screening of a series of natural plant compounds in TGEV-infected porcine intestinal epithelial cells and identified quercetin, a plant-derived flavonoid, as a potent antiviral candidate. Quercetin significantly alleviated TGEV-induced cytopathic effects and reduced viral load, without directly inactivating viral particles. Interestingly, TGEV infection triggered excessive activation of PINK1/Parkin-mediated mitophagy, leading to mitochondrial membrane potential loss, mitochondrial reactive oxygen species (mtROS) accumulation, and suppression of respiratory chain components, which subsequently activated the NF-κB and JAK/STAT signaling pathways. However, quercetin restored mitochondrial function by suppressing mitophagy overactivation, preserving mitochondrial membrane potential and mtDNA levels, and attenuating oxidative stress. Moreover, functional interference assays revealed that the anti-inflammatory efficacy of quercetin was dependent on its ability to maintain mitochondrial homeostasis and inhibit pathological mitophagic flux. These findings were validated in a TGEV-infected piglet model, where excessive mitophagy correlated closely with intestinal inflammation signaling activation. Collectively, our results not only indicated a novel mechanism of mitophagy-driven mitochondrial dysfunction in TGEV pathogenesis, but also suggested that quercetin may serve as a potential mitochondria-targeted natural compound for mitigating coronavirus-induced intestinal inflammation.

## Introduction

Coronaviruses are a group of highly contagious RNA viruses with broad host ranges and interspecies transmission capabilities 1-3. In recent years, they have caused repeated global public health emergencies, posing a continuous threat to human health and severely impacting livestock production 4,5. Transmissible gastroenteritis virus (TGEV), an α-coronavirus widely circulating in swine populations, primarily targets intestinal epithelial cells of neonatal piglets, leading to severe diarrhea, dehydration, and high mortality, thus significantly hindering the development of the swine industry 6. TGEV is mainly transmitted via the fecal-oral route, infecting and replicating in the villous epithelial cells of the small intestine—especially the jejunum and ileum—where it disrupts the intestinal barrier and triggers robust immune responses 7,8. Therefore, TGEV serves not only as an important model for studying the pathogenic mechanisms of intestinal viruses but also provides a valuable basis for understanding coronavirus-induced intestinal inflammation and host immune dysregulation.

Intestinal epithelial cells (IECs), the primary targets of TGEV infection, play essential roles in maintaining gut barrier integrity and regulating mucosal immune responses 9,10. Previous studies have demonstrated that TGEV disrupts epithelial structure and function, leading to the production of proinflammatory cytokines such as IL-1β, IL-6, and TNF-α, and resulting in epithelial injury 11,12. Recently, mitochondria have emerged as key regulators in viral pathogenesis, functioning at the intersection of cellular metabolism, redox control, and immune signaling 13,14. Mitochondrial dysfunction, including membrane potential collapse, excessive ROS, and bioenergetic failure, can activate inflammatory cascades that worsen tissue damage 15-17. To maintain mitochondrial homeostasis, cells employ mitophagy, a selective autophagic process mediated primarily through the PINK1/Parkin pathway, to eliminate damaged mitochondria 18. While this mechanism is protective under physiological conditions, excessive mitophagy may paradoxically deplete mitochondrial reserves and contribute to immune dysregulation. Whether TGEV infection perturbs this quality control system to promote epithelial inflammation remains unknown. Elucidating the mitochondrial-inflammatory axis in TGEV-infected IECs is therefore critical for understanding virus-induced intestinal pathology.

Given the pivotal role of mitochondrial dysfunction and inflammation in viral pathogenesis 19, increasing efforts have focused on natural plant compounds capable of targeting these processes. In RNA virus infection models, plant-derived bioactive molecules have shown promise in restoring mitochondrial function, alleviating oxidative stress, and modulating host immune responses 20. For instance, silibinin (a flavonolignan complex) has shown *in vivo* antiviral efficacy against HCV in patients unresponsive to standard therapy 21. Quercetin, another flavonoid, demonstrated stronger inhibition of HSV-1 replication than acyclovir in comparative studies 22. These findings prompted us to explore whether such compounds could confer protection against TGEV-induced cellular injury. To this end, we established a TGEV infection model using porcine intestinal epithelial cells (IPEC-J2) and systematically screened 20 representative plant extracts for antiviral activity *in vitro*. Several candidates were found to alleviate cytopathic effects, enhance cell viability, and reduce viral replication, among which quercetin exhibited the most pronounced efficacy. Quercetin, a flavonoid widely distributed in fruits and vegetables, has attracted considerable attention for its broad pharmacological activities, including anti-inflammatory and antioxidant properties 23,24. Although quercetin's immunomodulatory effects have been demonstrated in various disease contexts, its specific role in coronavirus-induced intestinal pathology remains undefined. In particular, whether quercetin modulates mitochondrial homeostasis and inflammatory responses during TGEV infection has yet to be elucidated.

In this study, we demonstrate that quercetin confers significant protection against TGEV-induced injury in intestinal epithelial cells. Mechanistically, TGEV infection triggered excessive activation of PINK1/Parkin-mediated mitophagy, resulting in mitochondrial membrane potential loss, mtDNA depletion, and elevated ROS levels, which collectively contributed to sustained proinflammatory cytokine production. Quercetin treatment effectively suppressed this mitophagic overactivation, preserved mitochondrial integrity, and attenuated downstream inflammatory signaling pathways. Notably, modulation of the mitophagy-mitochondrial function-inflammation axis emerged as a central mechanism underlying quercetin's anti-inflammatory activity. These findings uncover a previously unrecognized pathological cascade in TGEV infection, wherein dysregulated mitophagy drives mitochondrial dysfunction and immune activation. Our results provide mechanistic insight into virus-induced intestinal injury and support the potential role of quercetin as a mitochondria-targeted strategy that may help alleviate coronavirus-associated intestinal inflammation.

## Materials and Methods

### Cell Culture and Virus

The porcine intestinal epithelial cell line (IPEC-J2) was obtained from the American Type Culture Collection (ATCC) and cultured in DMEM/F12 (Gibco, Shanghai, China; 11320033) supplemented with 10% fetal bovine serum (Gibco, Shanghai, China; 10099141C) and 1% penicillin-streptomycin at 37 °C in a 5% CO₂ atmosphere. The TGEV strain TS (GenBank accession no. DQ201447.1) was kindly provided by the College of Veterinary Medicine, Sichuan Agricultural University and propagated in IPEC-J2 cells.

### Animals

The initial *in vivo* experiment was conducted using our previously established TGEV-infected piglet model, as described in our earlier work 25, to investigate whether TGEV infection induces inflammatory responses and PINK1/Parkin-mediated mitophagy in intestinal tissues. A total of 12 castrated male Duroc × Landrace × Yorkshire (DLY) piglets with similar body weight were randomly assigned into two groups: (1) control group receiving oral culture medium and (2) TGEV-infected group receiving 2.8 × 10⁹ PFU of TGEV via oral gavage at 5 weeks of age.

To further examine the protective effect of quercetin, a second *in vivo* experiment was performed with 15 castrated male DLY piglets of similar body weight. Animals were randomly divided into three groups: (1) control, (2) TGEV-infected, and (3) TGEV + quercetin. Animals in the quercetin group received quercetin mixed into the feed at a dosage of 200 mg/kg diet, starting 14 days prior to viral challenge and continuing until the end of the experiment. All groups were infected with TGEV at 5 weeks of age. Piglets were humanely euthanized with sodium pentobarbital. Following euthanasia, approximately 3-4 cm mid-jejunum segments were excised at a uniform location, rinsed with ice-cold PBS, and either snap-frozen in liquid nitrogen for assays.

All animal procedures were approved by the Institutional Animal Care and Use Committee of Sichuan Agricultural University (Approval No. SICAU-2015-018).

### Reagents

A total of 20 plant-derived extracts were obtained from Guilin Layn Natural Ingredients Corp, Ltd. Each extract was provided in powder form and dissolved in DMSO for *in vitro* experiments. Details of plant-derived compounds appear in Table S1 in Supporting Information.

### Cell viability assay and Live/dead cell staining

IPEC-J2 cells were plated in 96-well plates for culture and various treatments. After treatment with different compounds or viruses as indicated, 10 μL of Cell Counting Kit-8 (CCK-8, Beyotime, Shanghai, China; C0038) solution was added to each well and incubated at 37 °C for 2 h in the dark. The absorbance was then measured at 450 nm using a microplate reader (SpectraMax M2, Molecular Devices, USA), and cell viability was calculated relative to the untreated control group.

To evaluate cell viability and membrane integrity, a Calcein-AM/Propidium Iodide (PI) double staining assay was performed using a commercial viability kit (Beyotime, Shanghai, China; C2015M) following the manufacturer's instructions. After treatment, cells were gently washed with PBS and incubated with a mixed staining solution containing Calcein-AM and PI at 37 °C for 30 minutes in the dark. Subsequently, cells were rinsed and immediately imaged using a fluorescence microscope. Live cells exhibited green fluorescence (Calcein-AM), while dead or membrane-damaged cells showed red fluorescence (PI).

### Quantitative Real-Time PCR

Total RNA was extracted from intestinal tissues or cultured cells using RNAiso Plus (Takara Bio, Dalian, China; 9109) according to the manufacturer's instructions. The concentration and purity of RNA were assessed by NanoDrop spectrophotometry (Thermo Fisher Scientific, Waltham, MA, USA), and RNA integrity was verified by agarose gel electrophoresis. One microgram of total RNA was reverse-transcribed into cDNA using PrimeScript™ RT Master Mix (Takara Bio, Dalian, China; RR036A).

qPCR was performed using TB Green® Premix Ex Taq™ II (Takara Bio, Dalian, China; RR820A) on a QuantStudio 6 Real-Time PCR System (Thermo Fisher Scientific, Waltham, MA, USA). Each reaction (10 μL) contained 5 μL TB Green Premix, 0.4 μL forward primer (10 μM), 0.4 μL reverse primer (10 μM), 1 μL cDNA template, and 3.2 μL nuclease-free water. The cycling protocol was as follows: initial denaturation at 95 °C for 30 s, followed by 40 cycles of 95 °C for 5 s and 60 °C for 30 s.

β-actin was used as the reference gene for normalization, and relative mRNA expression levels were calculated using the 2^-ΔΔCt method. All primers were designed to span exon-exon junctions when possible, synthesized by Sangon Biotech (Shanghai, China), and are listed in Table S2 along with amplicon size.

### Western blotting

Western blotting was performed following established protocols with minor modifications 26. Intestinal tissues or cultured cells were rinsed with PBS and lysed using RIPA buffer (Solarbio, Beijing, China; R0010). Protein concentrations were quantified using a BCA protein assay kit (Thermo Fisher, Waltham, MA, USA; 23225) according to the manufacturer's instructions. Equal amounts of protein samples were separated by SDS-PAGE and transferred onto PVDF membranes. Membranes were blocked with 5% BSA in TBST (Tris-buffered saline with 0.1% Tween-20) for 1 hour at room temperature, followed by overnight incubation with primary antibodies at 4 °C. After washing, membranes were incubated with HRP-conjugated secondary antibodies for 1 hour at room temperature. Immuno-reactive proteins were visualized using a chemiluminescence detection kit (Beyotime, Shanghai, China; P0018FS). Protein bands were visualized and quantified using the ChemiDoc™ Touch Imaging System (Bio-Rad, Hercules, CA, USA), and band densities were analyzed using Image Lab software (v6.1) to determine relative protein expression. Antibody details are listed in Table S1.

### Preparation of Jejunal Single-Cell Suspensions for Flow Cytometry

To assess cellular responses in jejunal epithelial tissue, intestinal samples were processed into single-cell suspensions following previously described protocols with minor modifications 27. Briefly, freshly excised jejunum tissues were diced and incubated in 10 mL of Hanks' Balanced Salt Solution containing dithiothreitol at 37 °C for 30 minutes to remove mucus. The tissue was then transferred to 10 mL of HBSS containing 0.5 mM EDTA and shaken at 37 °C for an additional 30 minutes to facilitate epithelial cell dissociation. After discarding the supernatant, the remaining tissue was digested in 1 mg/mL collagenase A and 50 ug/mL DNase I at 37 °C for 30 min with gentle agitation. The resulting suspension was filtered through a 70 μm cell strainer (Absin, Shanghai, China; abs7232) to obtain a single-cell suspension and used immediately for downstream flow cytometry analysis.

### TGEV and p-P65 Detection

To detect TGEV-infected and p-P65-positive cells, cells were collected and washed once with PBS, followed by fixation in 4% paraformaldehyde for 15 minutes at room temperature. After centrifugation (500 × g, 5 min), cells were permeabilized with 0.05% Triton X-100 for 10 minutes and incubated with primary antibodies against the TGEV nucleocapsid protein (1:100, diluted in 0.05% Triton X-100/PBS) and phospho-NF-κB p65 (Ser536) (1:100, diluted in 0.05% Triton X-100/PBS) for 2 hours at room temperature. After washing, cells were incubated with corresponding fluorophore-conjugated secondary antibodies (1:1000 in PBS) for 1 hour in the dark. Finally, cells were washed again, resuspended in 500 μL PBS, and analyzed by flow cytometry.

### Mitochondrial Functional Analysis

To evaluate mitochondrial status under different treatment conditions, the following fluorescent probes were applied: MitoSOX™ Red (MedChemExpress, NJ, USA; HY-D1055): A mitochondrial superoxide indicator used to detect mtROS. Cells were incubated with 5 μM MitoSOX Red at 37 °C for 30 min in the dark, washed twice with PBS, and immediately analyzed by flow cytometry (Ex/Em: 510/580 nm). Rhodamine 123 (MedChemExpress, NJ, USA; HY-D0816): Used for assessing mitochondrial membrane potential. Cells were incubated with 5 μM Rhodamine 123 for 30 min at 37 °C in the dark, washed gently with PBS, and analyzed by flow cytometry (Ex/Em: 507/529 nm). MitoTracker™ Deep Red FM (Beyotime, Shanghai, China; C1032): Applied to assess mitochondrial biomass. Cells were stained with 200 nM MitoTracker Deep Red FM for 30 min at 37 °C, washed with PBS, and analyzed by flow cytometry (Ex/Em: 644/665 nm).

### Immunofluorescence

Cells were fixed with 4% paraformaldehyde for 15 min at room temperature, permeabilized with 0.1% Triton X-100 for 10 min, and incubated with primary antibodies overnight at 4 °C. After washing, cells were treated with fluorescent secondary antibodies for 1 h in the dark, followed by DAPI staining (Beyotime, Shanghai, China; P0131) for 10 min. Images were acquired using a Leica DMI4000B inverted fluorescence microscope (Leica Microsystems, Wetzlar, Germany) or FV4000 confocal microscope (Evident, Tokyo, Japan).

Fluorescent Probe and Co-localization Assays: For mitochondrial ROS detection, cells were incubated with MitoSOX™ Red for 30 min at 37 °C in the dark. In LC3 flux analysis, IPEC-J2 cells transfected with mCherry-GFP-LC3 were observed for red/green fluorescence patterns. For mitophagy co-localization, cells were co-stained with MitoTracker and LysoTracker, followed by imaging to assess mitochondrial-lysosome co-localization. All images were captured using a Leica DMI4000B inverted fluorescence microscope or FV4000 confocal microscope.

### Bioinformatics Analysis of TGEV-Related Targets and Quercetin Intersections

To identify potential target genes of quercetin in the context of TGEV infection, we first identified differentially expressed genes (DEGs) from the GSE182240 transcriptome dataset using the “limma” package in R. In parallel, 693 TGEV-related genes were retrieved from the GeneCards database. These two datasets (DEGs and GeneCards) were merged to generate a pool of virus-associated, expression-altered genes. We then intersected this combined gene set with 208 quercetin-related targets obtained from the Traditional Chinese Medicine Systems Pharmacology (TCMSP) database. This analysis yielded 82 overlapping genes, which were used for PPI network construction and GO/KEGG enrichment analysis.

### Molecular Docking

Molecular docking was conducted with reference to previously described methods 28, with appropriate adjustments to fit our target-compound context. The 3D structure of quercetin was downloaded from the PubChem database. Protein structures for NFκBIA, TNFA, and STAT1 were retrieved from the RCSB Protein Data Bank. Molecular docking was performed using AutoDock Vina software to evaluate the binding affinity between quercetin and inflammatory regulators. Binding poses were visualized using PyMOL.

### Statistical Analysis

All experiments were performed in at least three independent biological replicates. Data are expressed as mean ± SEM. Flow cytometry data were analyzed using FlowJo (version 10.8.1). Correlation analyses were performed using Pearson's correlation coefficient. Diarrhea incidence was analyzed using generalized estimating equations (GEE, binomial logit link) with pig ID as a clustering factor; in cases of complete separation, Fisher's exact test was applied. Prior to statistical analyses, data normality was assessed using the Shapiro-Wilk test, and homogeneity of variances was examined using Levene's test (GraphPad Prism 9.0). For comparisons between two groups, the unpaired two-tailed Student's t-test was used. For multiple group comparisons, one-way ANOVA followed by Tukey's post-hoc test was applied. A p-value of < 0.05 was considered statistically significant.

## Results

### Quercetin identified as an effective antiviral candidate against TGEV from plant-derived extracts

To explore the antiviral potential of natural plant-derived compounds against TGEV, we screened twenty representative extracts using a TGEV-infected IPEC-J2 cell model. A combination of cytotoxicity assessment, morphological observation, cell viability analysis, and viral load quantification was used to evaluate their protective efficacy. We first assessed the cytotoxicity of these compounds in IPEC-J2 cells and selected the highest concentration that did not affect cell viability for subsequent experiments (Figure S1). Consistent with hallmark features of TGEV infection, infected untreated IPEC-J2 cells exhibited pronounced morphological damage and structural disintegration (Figure S2A). Among all tested candidates, procyanidin and quercetin markedly attenuated TGEV-induced cytopathic effects, indicating their potential antiviral efficacy.

**Figure 1:**
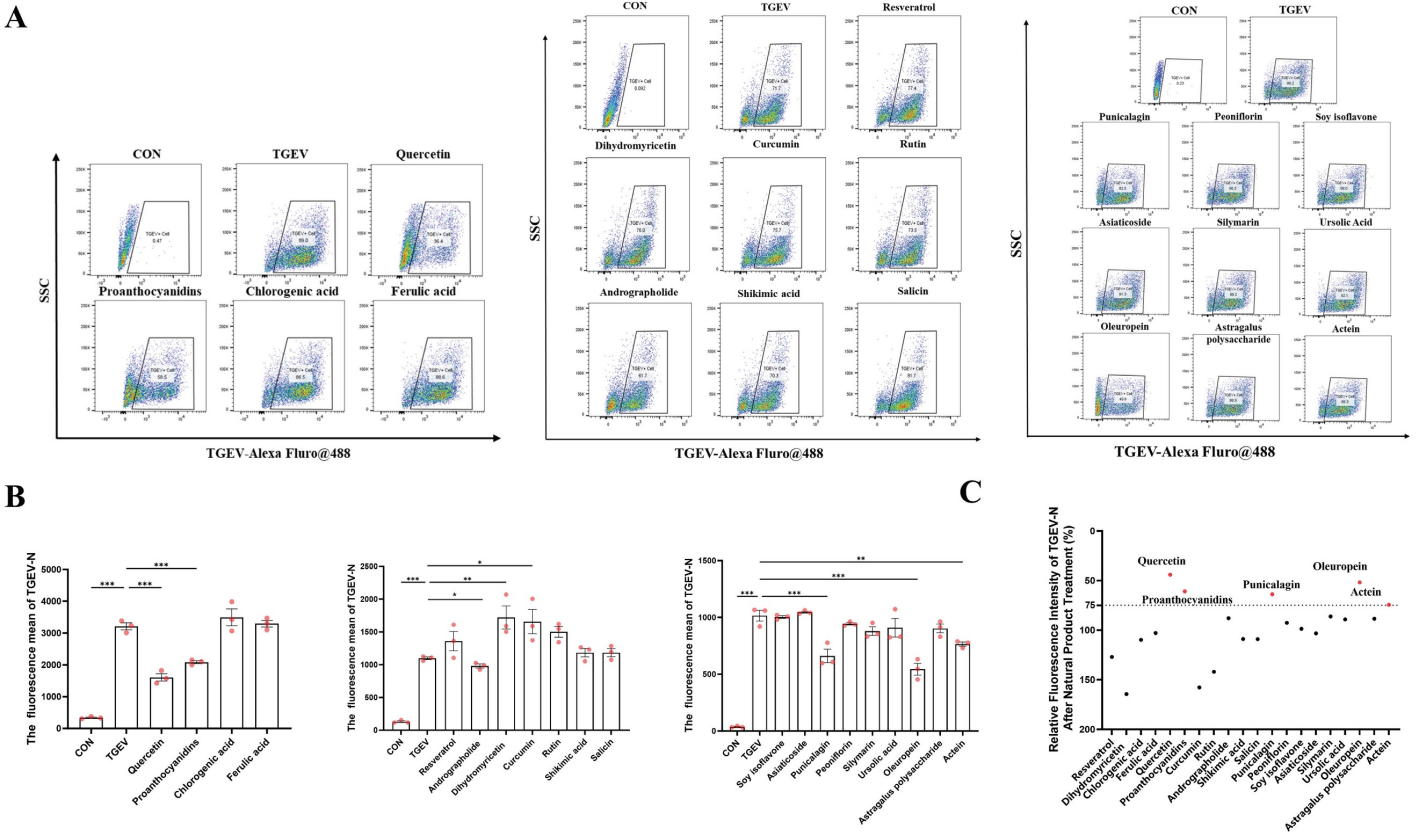
** Screening and evaluation of natural plant compounds for antiviral effects against TGEV in IPEC-J2 cells.** IPEC-J2 cells were pretreated with 20 natural plant compounds for 1 h, followed by TGEV infection (MOI = 1) for 24 h. The working concentrations of the tested compounds were: shikimic acid (100 μg/mL), chlorogenic acid (100 μg/mL), rutin (100 μg/mL), curcumin (5 μM), salicin (100 μg/mL), dihydromyricetin (50 μM), resveratrol (25 μg/mL), andrographolide (100 μg/mL), proanthocyanidins (40 μM), quercetin (80 μM), ferulic acid (100 μg/mL), punicalagin (6.25 μg/mL), peoniflorin (100 μg/mL), soy isoflavone (100 μg/mL), actein (100 μg/mL), astragalus polysaccharide (100 μg/mL), asiaticoside (100 μg/mL), silymarin (25 μg/mL), ursolic acid (6.25 μg/mL), and oleuropein (100 μg/mL). (A) Flow cytometric analysis of intracellular TGEV-N protein using Alexa Fluor® 488-labeled antibody. Representative dot plots of each treatment group (n = 3). (B) Quantification of mean fluorescence intensity (MFI) of intracellular TGEV-N protein in selected treatment groups, reflecting antiviral efficiency (n = 3). (C) Relative fluorescence intensity of TGEV-N after treatment with different natural products, expressed as a percentage relative to the virus control group (TGEV). Top-performing compounds are highlighted in red. Data were expressed as mean ±SEM. ^*^p < 0.05, ^**^p < 0.01, ^***^p < 0.001.

We next examined cell viability post-infection and treatment. Quercetin, procyanidin, soy isoflavones, and oleuropein significantly restored cell viability in TGEV-infected cells (*p* < 0.01) (Figure S2B), suggesting their ability to counteract virus-induced cytotoxicity. To further assess antiviral efficacy, we quantified intracellular viral load using flow cytometry. Several compounds—including quercetin, procyanidin, andrographolide, punicalagin, and oleuropein—significantly reduced viral load We next examined cell viability post-infection and treatment. Quercetin, procyanidin, soy isoflavones, and oleuropein significantly restored cell viability in TGEV-infected cells (*p* < 0.05) (Figure S2B), suggesting their ability to counteract virus-induced cytotoxicity. To further assess antiviral efficacy, we quantified intracellular viral load using flow cytometry. Several compounds—including quercetin, procyanidin, andrographolide, punicalagin, and oleuropein—significantly reduced viral load (*p* < 0.05) (Figure 1A-C). Notably, quercetin and procyanidin consistently demonstrated strong protective effects across all evaluation parameters. Based on these integrated results, quercetin was selected as the primary candidate for further mechanistic studies due to its potent antiviral activity and favorable cytoprotective profile (Figure 1A-B).

Notably, quercetin and procyanidin consistently demonstrated strong protective effects across all evaluation parameters (Figure 1C). Based on these integrated results, quercetin was selected as the primary candidate for further mechanistic studies due to its potent antiviral activity and favorable cytoprotective profile.

### Effects of quercetin against TGEV infection and target network-based mechanistic insights

**Figure 2:**
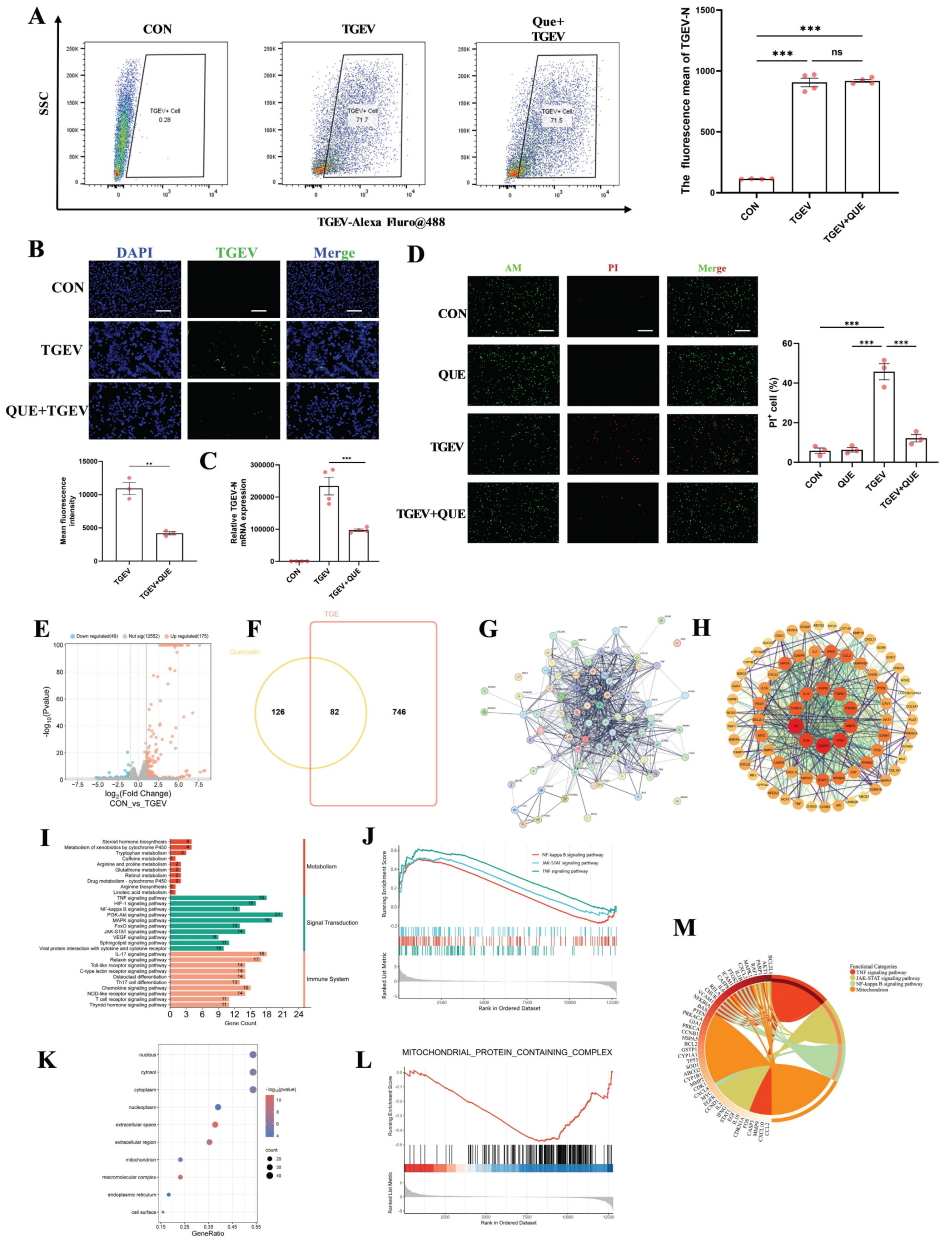
** Effects of quercetin against TGEV infection and target network-based mechanistic insights.** Unless otherwise specified, IPEC-J2 cells were pretreated with quercetin (80 μM) for 1 h before infection with TGEV (MOI = 1) for 24 h. (A) Flow cytometric analysis of intracellular TGEV-N protein in IPEC-J2 cells infected with TGEV for 24 h. The virus was pre-incubated with or without quercetin (80 μM) at 4 °C for 24 h prior to cell infection (n = 3). (B) Immunofluorescence staining and quantification of intracellular TGEV-N protein (green) and nuclei (DAPI, blue) in IPEC-J2 cells treated under standard quercetin pretreatment conditions (n = 3). Scale bar = 100 μm. (C) Quantitative RT-PCR analysis of *TGEV-N* mRNA levels in IPEC-J2 cells following quercetin treatment and TGEV infection (n = 4). (D) Live/dead staining using Calcein-AM (green) and propidium iodide (PI, red) in IPEC-J2 cells (n = 3). Scale bar = 100 μm. (E) Volcano plot showing differentially expressed genes (DEGs) in TGEV-infected intestinal organoids from GEO dataset GSE182240 (F) Venn diagram illustrating 82 overlapping genes among TGEV-related genes (GeneCards), quercetin targets (TCMSP), and DEGs from GSE182240. (G) Protein-protein interaction (PPI) network of the overlapping genes constructed using the STRING database. (H) Compound-target-pathway network visualizing quercetin and its putative host targets in the context of TGEV infection. (I) KEGG pathway enrichment analysis of the overlapping genes. (J) Gene Set Enrichment Analysis (GSEA) of transcriptomic data (GSE182240) showing enrichment of inflammatory signaling pathways including NF-κB, TNF, and JAK-STAT pathways in TGEV-infected organoids (K) GO cellular component enrichment. (L) GSEA analysis indicating suppression of mitochondrial protein-containing complex-related genes in TGEV-infected organoids. (M) Chord diagram depicting associations between GO terms and core target genes. Data were expressed as mean ±SEM. ^**^p < 0.01, ^***^p < 0.001.

To further validate the antiviral effect of quercetin against TGEV infection, we designed two experimental approaches. In the first approach, quercetin was pre-incubated with TGEV at 4 °C for 24 hours prior to infecting IPEC-J2 cells. Flow cytometric analysis of intracellular TGEV-N protein levels revealed no significant reduction (Figure 2A), suggesting that quercetin does not directly inactivate the virus. In contrast, when quercetin was directly added to IPEC-J2 cells under normal culture conditions, subsequent immunofluorescence analysis showed a marked decrease in viral load and a significant increase in cell viability (*p* < 0.01) (Figure 2B-D), supporting a host-mediated protective mechanism.

To explore the potential molecular targets involved, we analyzed the transcriptomic dataset GSE182240 from the GEO database, which profiled TGEV-infected intestinal organoids. A total of 175 upregulated and 46 downregulated genes were identified (Figure 2E). Additionally, we retrieved 693 TGEV-related genes from the GeneCards database and 208 quercetin-associated targets from the TCMSP platform. Cross-referencing these datasets yielded 82 overlapping genes potentially involved in both quercetin action and TGEV response (Figure 2F). A protein-protein interaction (PPI) network constructed using the STRING database revealed key hub genes, including IL6, TNFα, and STAT3 (Figure 2G-H).

Gene Ontology (GO) and KEGG enrichment analyses indicated that the potential targets of quercetin were significantly involved in pro-inflammatory signaling pathways, including TNFα, NF-κB, and JAK/STAT pathway (Figure 2I). GSEA analysis validated positive enrichment of canonical inflammatory pathways including NF-κB, TNF, and JAK-STAT signaling (Figure 2J). GO analysis showed a predominant enrichment of these targets in the nucleus and mitochondria (Figure 2K), suggesting that quercetin may modulate cellular stress responses during infection, a notion further supported by GSEA-based downregulation of mitochondria-associated genes (Figure 2L). Moreover, chord diagram visualization was employed to illustrate the complex interactions between core genes and their associated functional categories, providing an intuitive overview of the multifunctional roles of quercetin-related targets in antiviral and cellular regulatory processes (Figure 2M).

### TGEV infection induces mitochondrial dysfunction and inflammatory signaling activation

**Figure 3:**
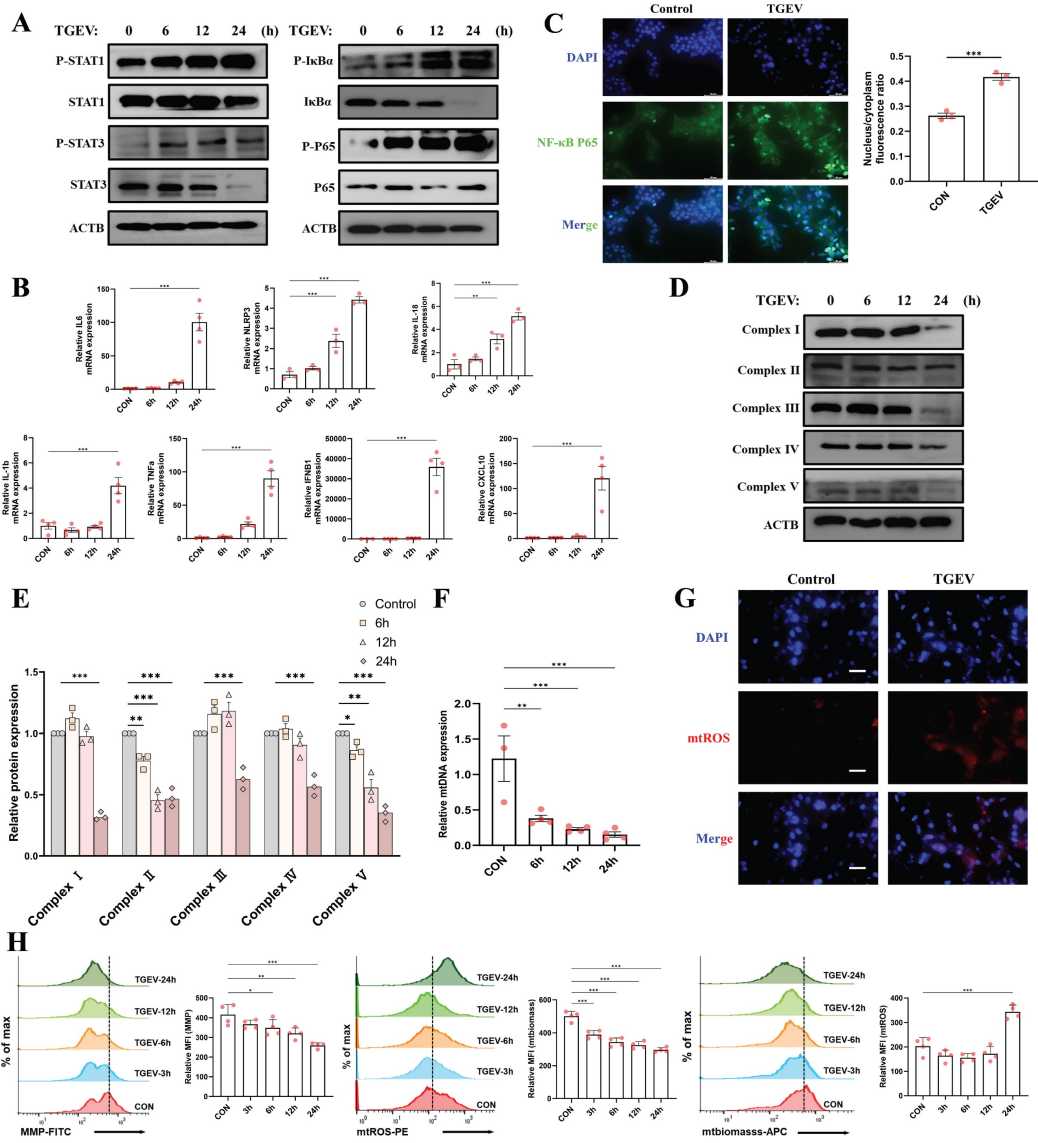
** TGEV infection induces mitochondrial dysfunction and inflammatory signaling activation.** IPEC-J2 cells were infected with TGEV (MOI = 1) for 0, 6, 12, and 24 hours to assess time-dependent changes in inflammation and mitochondrial function. (A) Western blot analysis of phosphorylated and total levels of STAT1, STAT3, IκBα, and NF-κB p65 at indicated time points post-infection. (B) Quantitative RT-PCR analysis of mRNA expression of pro-inflammatory cytokines (*IL-1β*, *IL-6*, *IL-18*, *TNF-α*), interferon-stimulated genes (*IFNB1*, *CXCL10*), and *NLRP3* at 24 hours post-infection. (n = 3-4) (C) Immunofluorescence staining of NF-κB p65 (green) and DAPI (blue) showing subcellular distribution of NF-κB p65 in control and TGEV-infected cells. Quantification of nuclear/cytoplasmic ratio is shown on the right. Scale bar = 50 μm. (D-E) Western blot analysis of mitochondrial electron transport chain complexes I-V at various time points post-infection. (F) Quantification of relative mtDNA copy number by qPCR following TGEV infection (n = 3-4). (G) Immunofluorescence staining of mtROS (red) and nuclei (DAPI, blue) in control and TGEV-infected cells. Scale bar = 50 μm. (H) Flow cytometric analysis of mitochondrial health indicators, including mitochondrial membrane potential, mtROS, and mitochondrial biomass at various time points (3, 6, 12, and 24 h) following infection (n = 3-4). Data were expressed as mean ±SEM. ^*^p < 0.05, ^**^p < 0.01, ^***^p < 0.001.

Building on earlier bioinformatics analysis suggesting the enrichment of mitochondrial- and inflammation-related genes among quercetin-TGEV overlapping targets, we next evaluated the dynamic changes in inflammatory signaling and mitochondrial function following TGEV infection. Western blot analysis revealed early and progressive activation of both the NF-κB and JAK-STAT pathways. Phosphorylation of STAT1, STAT3, IκBα, and NF-κB p65 was detected as early as 6 hours post-infection (hpi) and peaked at 24 hpi (Figure 3A). qPCR further confirmed robust upregulation of pro-inflammatory cytokines (*IL-1β*, *IL-6*, *IL-18*, *TNF-α*), interferon-stimulated genes (*IFNB1*, *CXCL10*), and *NLRP3* at 24 hpi (*p* < 0.001) (Figure 3B). Immunofluorescence staining also showed increased nuclear localization of NF-κB p65 in infected cells (*p* < 0.001) (Figure 3C), indicating transcriptional activation of pro-inflammatory genes.

To further investigate mitochondrial status, we assessed both protein-level and functional indicators of mitochondrial health. Western blot showed progressive downregulation of mitochondrial respiratory chain complexes I-V (*p* < 0.05) (Figure 3D-E), while qPCR revealed reduced mtDNA copy number following infection (*p* < 0.01) (Figure 3F). These results suggest impaired mitochondrial integrity upon TGEV challenge. Moreover, immunofluorescence and flow cytometry analyses demonstrated that TGEV infection led to significant elevation of mtROS (Figure 3G), along with reduced mitochondrial membrane potential (MMP) and biomass (*p* < 0.05) (Figure 3H). These disruptions reflect mitochondrial depolarization and oxidative stress, indicative of compromised mitochondrial homeostasis.

Together, these findings suggest that TGEV infection triggers concurrent activation of inflammatory signaling and disruption of mitochondrial integrity. Given the well-established crosstalk between mitochondrial function and innate immunity, the mechanistic link between these two processes warrants further investigation.

### Quercetin alleviates TGEV-induced inflammation and mitochondrial damage

**Figure 4:**
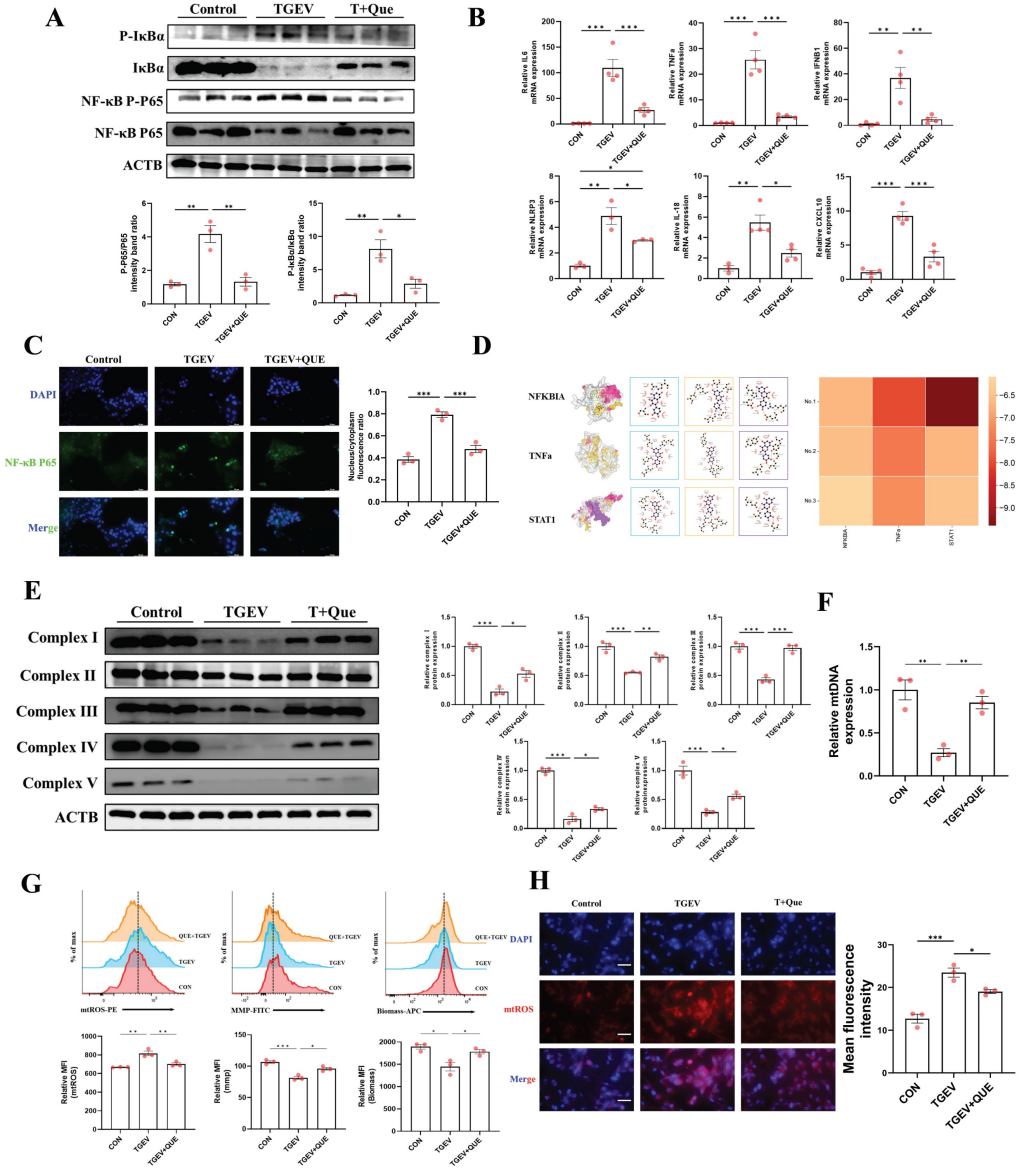
** Quercetin alleviates TGEV-induced inflammation and mitochondrial damage.** IPEC-J2 cells were pretreated with quercetin (80 μM) for 1 h before infection with TGEV (MOI = 1) for 24 h. (A) Western blot analysis of phosphorylated and total levels of IκBα and NF-κB p65 in each group. (B) Quantitative RT-PCR analysis of mRNA expression of *TNF-α*, *IL-6*, *IL-1β*, *NLRP3*, *IFNB1*, and *CXCL10* (n = 3-4). (C) Immunofluorescence staining of NF-κB p65 (green) and nuclei (DAPI, blue) to assess p65 nuclear translocation. Scale bar = 50 μm. (D) Molecular docking analysis of quercetin with three inflammation-related proteins (NFκBIA, TNFA, STAT1). (E) Western blot analysis of mitochondrial electron transport chain complexes I-V. (F) Quantification of mtDNA copy number by qPCR in each group (n = 3). (G) Flow cytometric analysis of MMP, mitochondrial biomass, and mtROS (n = 3). (H) Immunofluorescence staining of mtROS (red) and nuclei (DAPI, blue) in IPEC-J2 cells. Scale bar = 50 μm. Data were expressed as mean ±SEM. ^*^p < 0.05, ^**^p < 0.01, ^***^p < 0.001.

As TGEV infection was shown to induce robust inflammatory signaling and mitochondrial dysfunction (Figure 3), we next tested whether quercetin could mitigate these effects. As shown by Western blot and qPCR, quercetin treatment markedly inhibited the activation of inflammatory pathways triggered by TGEV infection (*p* < 0.05) (Figure 4A-B). Specifically, quercetin suppressed NF-κB pathway activation, as evidenced by reduced phosphorylation levels of IκBα and p65. This was accompanied by downregulation of proinflammatory cytokine mRNA levels, including *TNF-α*, *IL-6*, and *IL-1β*, as well as *NLRP3* (*p* < 0.05), an inflammasome component often implicated in viral inflammation 29,30 (Figure 4B). Furthermore, quercetin also dampened the expression of *IFNB1* and *CXCL10* (*p* < 0.01), implicating a modulatory effect on the JAK-STAT/interferon signaling cascade (Figure 4B). Immunofluorescence revealed that quercetin suppressed TGEV-induced nuclear translocation of NF-κB p65 (*p* < 0.001), indicating inhibition of its transcriptional activation (Figure 4C). To gain molecular insight into quercetin's potential targets, we conducted molecular docking simulations between quercetin and three core inflammation-related proteins—NFκBIA, TNFA, and STAT1. The top three binding conformations for each protein displayed binding free energies below -6.5 kcal/mol, indicating strong affinity. Since binding energies below -5.0 kcal/mol are typically considered indicative of meaningful interactions, these results suggest that quercetin may exert its immunomodulatory effects by directly interacting with these signaling mediators (Figure 4D).

We next explored whether quercetin could alleviate mitochondrial impairment induced by TGEV. Western blot analysis revealed that quercetin partially restored the expression of mitochondrial respiratory chain complexes I-V (*p* < 0.05), which were otherwise downregulated by infection (Figure 4E). Additionally, quercetin significantly recovered mtDNA copy number (*p* < 0.01), suggesting a protective role in mitochondrial biogenesis (Figure 4F). Flow cytometry showed that quercetin significantly reversed TGEV-induced MMP depolarization, restored mitochondrial biomass, and reduced the accumulation of mtROS, which was further confirmed by fluorescence microscopy (*p* < 0.05) (Figure 4G-H). Together, these findings indicate that quercetin effectively suppresses TGEV-induced inflammation and preserves mitochondrial integrity.

### Quercetin alleviates TGEV-induced inflammatory responses by improving mitochondrial function

**Figure 5:**
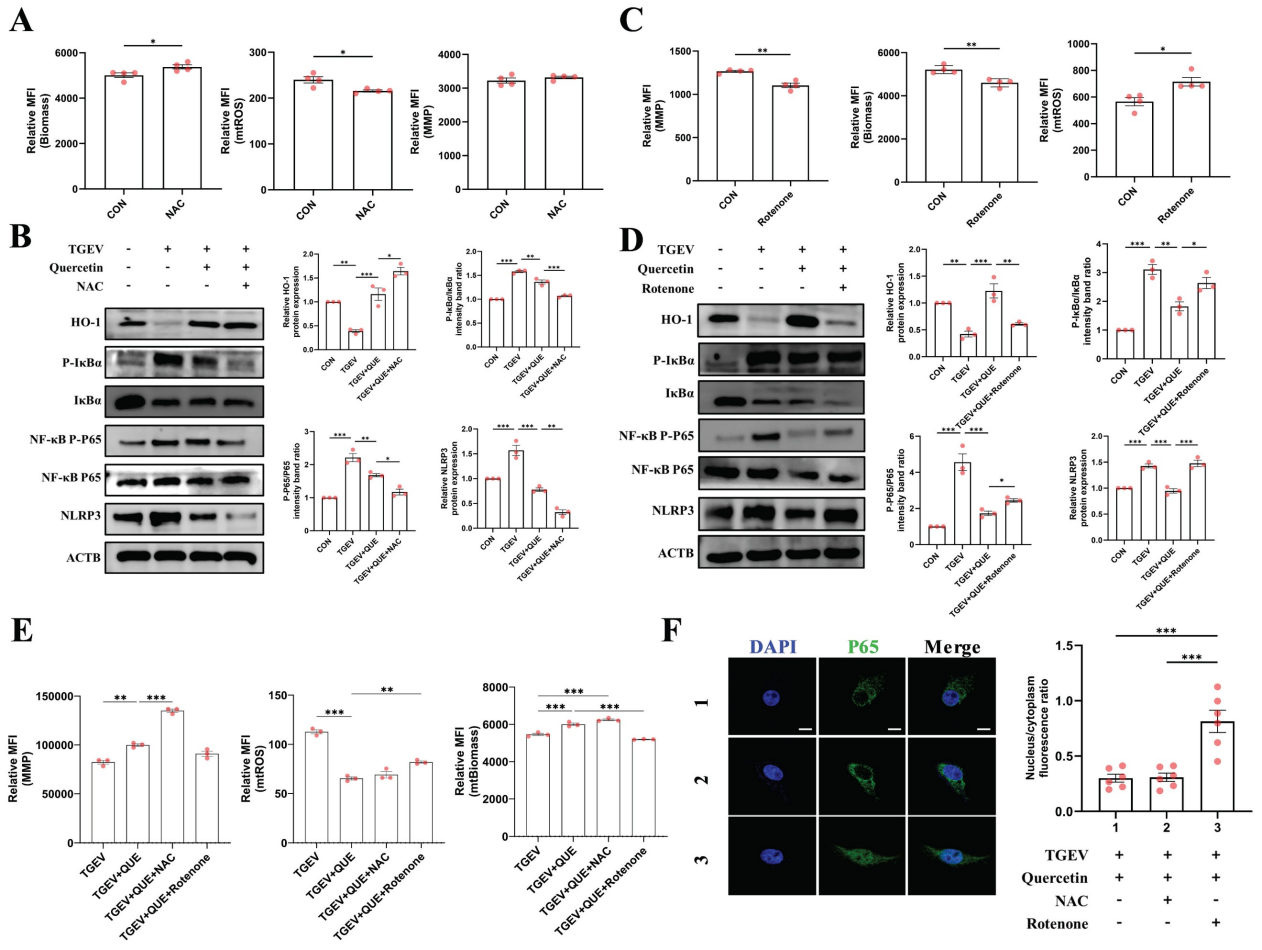
** Quercetin alleviates TGEV-induced inflammatory responses by improving mitochondrial function.** IPEC-J2 cells were pretreated with quercetin (80 μM) for 1 h and subsequently infected with TGEV (MOI = 1) for 24 h. To modulate mitochondrial function, cells were co-treated with either NAC (5 mM) or rotenone (1 μM). (A) Flow cytometric analysis of mitochondrial parameters following NAC treatment (n = 3). (B) Western blot analysis of phosphorylated IκBα, NF-κB p65, and NLRP3 in cells co-treated with quercetin and NAC. (C) Flow cytometric analysis of mitochondrial parameters following rotenone treatment (n = 3). (D) Western blot analysis of inflammatory signaling markers in cells co-treated with quercetin and rotenone. (E) Comparative flow cytometric assessment of MMP, mitochondrial biomass, and mtROS in cells treated with quercetin alone or in combination with NAC or rotenone (n = 3). (F) Immunofluorescence staining of NF-κB p65 (green) and nuclei (DAPI, blue), showing subcellular localization across different treatment groups. Scale bar = 5 μm. Data were expressed as mean ±SEM. ^*^p < 0.05, ^**^p < 0.01, ^***^p < 0.001.

Previous studies have suggested that mitochondrial status is closely associated to the regulation of inflammatory signaling, particularly under viral infection stress 31,32. Building on our own findings that quercetin alleviates both inflammation and mitochondrial dysfunction during TGEV infection, we hypothesized that mitochondrial homeostasis may play a critical regulatory role in modulating TGEV-induced inflammation. To examine this, we used N-acetylcysteine (NAC), a known enhancer of mitochondrial function, and rotenone, a mitochondrial complex I inhibitor that disrupts mitochondrial integrity, as functional tools to interfere with mitochondrial status during quercetin treatment. We first validated the effects of NAC and rotenone on mitochondrial parameters in IPEC-J2 cells. Flow cytometry analysis revealed that NAC significantly reduced mtROS levels (*p* < 0.05) without markedly altering MMP, but slightly enhanced mitochondrial biomass (*p* < 0.05) (Figure 5A). In contrast, rotenone treatment led to pronounced MMP depolarization, reduced mitochondrial biomass, and increased mtROS accumulation (*p* < 0.05) (Figure 5C), confirming its mitochondrial-disruptive capacity.

Next, we examined how modulation of mitochondrial function affected quercetin's ability to suppress TGEV-induced inflammation. Western blot analysis demonstrated that co-treatment with NAC further reduced the phosphorylation levels of IκBα and p65, as well as the expression of NLRP3, compared to quercetin alone (*p* < 0.05) (Figure 5B). Conversely, rotenone treatment attenuated quercetin's inhibitory effects on these inflammatory markers (*p* < 0.05) (Figure 5D). These findings suggest that the anti-inflammatory efficacy of quercetin is at least partially dependent on preserved mitochondrial function.

Flow cytometric analysis provided further mechanistic insights into the role of mitochondrial integrity in quercetin-mediated protection. As shown in Figure 5E, NAC co-treatment significantly enhanced quercetin's effects by further restoring MMP (*p* < 0.001). In contrast, rotenone markedly reversed these protective effects, evidenced by reduced biomass and elevated mtROS accumulation (*p* < 0.01). These data reinforce the conclusion that mitochondrial function is not merely correlated with, but likely required for, the anti-inflammatory action of quercetin during TGEV infection. Immunofluorescence analysis of NF-κB p65 nuclear translocation corroborated these results: rotenone re-induced p65 nuclear accumulation despite quercetin treatment, whereas NAC preserved cytoplasmic localization of p65 (Figure 5F). Collectively, these results support the hypothesis that quercetin-mediated attenuation of inflammation during TGEV infection is mechanistically linked to its ability to maintain mitochondrial homeostasis.

### Quercetin mitigates TGEV-induced inflammation by suppressing excessive PINK1/Parkin-dependent mitophagy

**Figure 6:**
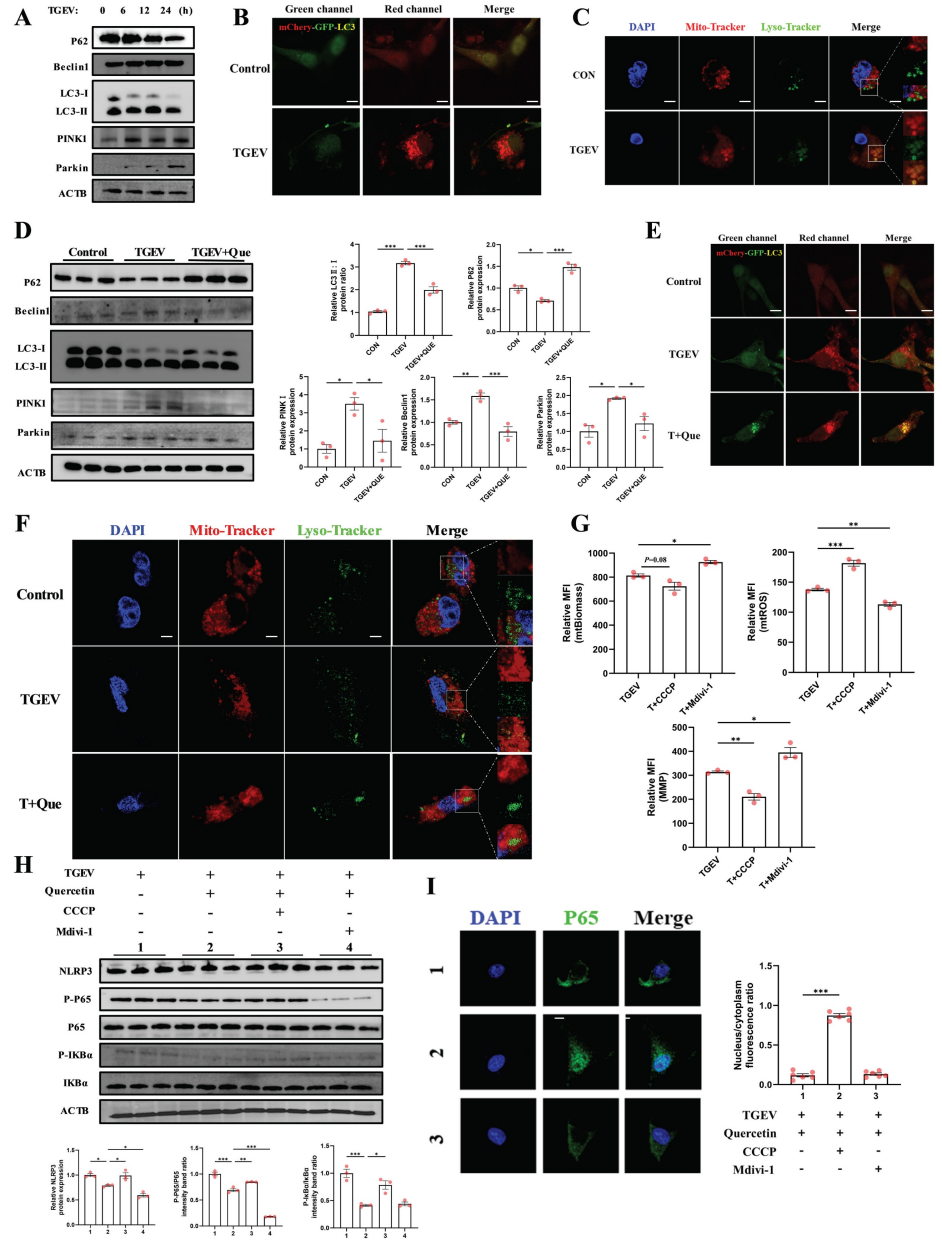
** Quercetin mitigates TGEV-induced inflammation by suppressing excessive PINK1/Parkin-dependent mitophagy.** IPEC-J2 cells were pretreated with quercetin (80 μM) for 1 h before infection with TGEV (MOI = 1) for 24 h. To modulate mitophagy, cells were treated with CCCP (5 μM) or Mdivi-1 (10 μM) as indicated. For autophagic flux detection, cells were infected with mCherry-GFP-LC3 adenovirus (MOI = 10) for 24 h prior to quercetin or virus exposure. (A) Western blot analysis of p62, Beclin1, LC3, PINK1, and Parkin at 0, 6, 12, and 24 h after TGEV infection. (B) Confocal imaging of IPEC-J2 cells infected with mCherry-GFP-LC3 adenovirus showing autophagosome (yellow) and autolysosome (red) signals following TGEV infection. Scale bar = 5 μm. (C) MitoTracker Red and LysoTracker Green co-staining with DAPI counterstaining in TGEV-infected cells to visualize mitochondrial-lysosome colocalization. Scale bar = 5 μm. (D) Western blot analysis of p62, LC3, PINK1, and Parkin in TGEV-infected cells treated with quercetin. (E) Confocal images of mCherry-GFP-LC3-expressing IPEC-J2 cells following quercetin treatment in TGEV-infected cells. Scale bar = 5 μm. (F) MitoTracker and LysoTracker co-staining in TGEV-infected cells treated with quercetin. Scale bar = 5 μm. (G) Flow cytometric analysis of MMP, mtROS, and mitochondrial biomass in TGEV-infected cells treated with quercetin, CCCP, or Mdivi-1 (n = 3). (H) Western blot analysis of phosphorylated p65, phosphorylated IκBα, and NLRP3 under different mitophagy modulation conditions. (I) Immunofluorescence staining of NF-κB p65 (green) and DAPI (blue) to assess nuclear translocation status across treatment groups. Scale bar = 5 μm. Data were expressed as mean ±SEM. ^*^p < 0.05, ^**^p < 0.01, ^***^p < 0.001.

We next asked whether mitophagy—a key regulator of mitochondrial quality control—might act as an upstream trigger of these pathogenic changes. Mitochondrial dysfunction is often accompanied by excessive mitophagy, a process that selectively eliminates damaged mitochondria to maintain cellular homeostasis 33. However, when excessively activated, mitophagy may lead to mitochondrial depletion and trigger inflammatory cascades 34. To investigate whether TGEV infection triggers mitophagy, we first examined the temporal expression of autophagy- and mitophagy-related markers. Western blot analysis showed that TGEV infection led to a time-dependent decrease in p62 and increased levels of Beclin1 and LC3-II/LC3-I ratio, indicative of activated autophagic flux. Simultaneously, the expression of PINK1 and Parkin was markedly elevated, suggesting induction of PINK1/Parkin-mediated mitophagy (Figure 6A). These findings were further validated using a mCherry-GFP-LC3 reporter system, which revealed enhanced red fluorescence upon TGEV infection, consistent with autolysosome formation (Figure 6B).

In addition, co-staining with Mito-Tracker and Lyso-Tracker demonstrated increased mitochondrial-lysosome colocalization in TGEV-infected cells (Figure 6C), confirming the activation of mitophagy.

Next, we assessed whether quercetin could mitigate TGEV-induced mitophagy. In quercetin-treated cells, p62 expression was restored and the LC3-II/LC3-I ratio was significantly reduced (Figure 6D), indicating suppression of autophagic flux. Notably, quercetin also decreased PINK1 and Parkin levels, further supporting its inhibitory effect on virus-induced mitophagy. Consistently, mCherry-GFP-LC3 fluorescence and Mito-Tracker/Lyso-Tracker co-localization were reduced following quercetin treatment (Figure 6E-F), reinforcing its role in limiting excessive mitophagy. These results collectively suggest that quercetin restores mitochondrial homeostasis by downregulating the PINK1/Parkin pathway.

To determine whether mitophagy contributes to quercetin's anti-inflammatory effects, we employed CCCP (a mitophagy inducer) and Mdivi-1 (a mitophagy inhibitor) as pharmacological tools. Flow cytometric analysis demonstrated that MMP and mitochondrial biomass were reduced, while mtROS levels were increased, in TGEV-infected cells treated with CCCP. In contrast, Mdivi-1 treatment partially restored MMP and biomass and reduced mtROS (*p* < 0.05) (Figure 6G). Immunoblotting showed that CCCP attenuated the inhibitory effects of quercetin on NF-κB p65 phosphorylation and NLRP3 expression (*p* < 0.05), while Mdivi-1 further potentiated these effects (*p* < 0.05) (Figure 6H). Immunofluorescence analysis of NF-κB p65 localization revealed that CCCP restored nuclear translocation of p65 in the presence of quercetin (*p* < 0.001), whereas Mdivi-1 further retained p65 in the cytoplasm (Figure 6I).

Taken together, these findings indicate that TGEV infection promotes excessive mitophagy through the PINK1/Parkin pathway, which contributes to mitochondrial dysfunction and inflammatory activation. Quercetin alleviates these effects by suppressing mitophagic flux, thereby stabilizing mitochondrial integrity and dampening downstream inflammatory responses.

### TGEV-induced mitophagy is closely associated with the activation of inflammatory signaling

Given the central role of mitophagy in mediating inflammation *in vitro* (Figure 6), we next sought to validate whether similar regulatory events occur *in vivo*. To this end, we analyzed intestinal tissues from TGEV-infected piglets to determine the status of PINK1/Parkin-mediated mitophagy and its association with inflammatory signaling.

**Figure 7:**
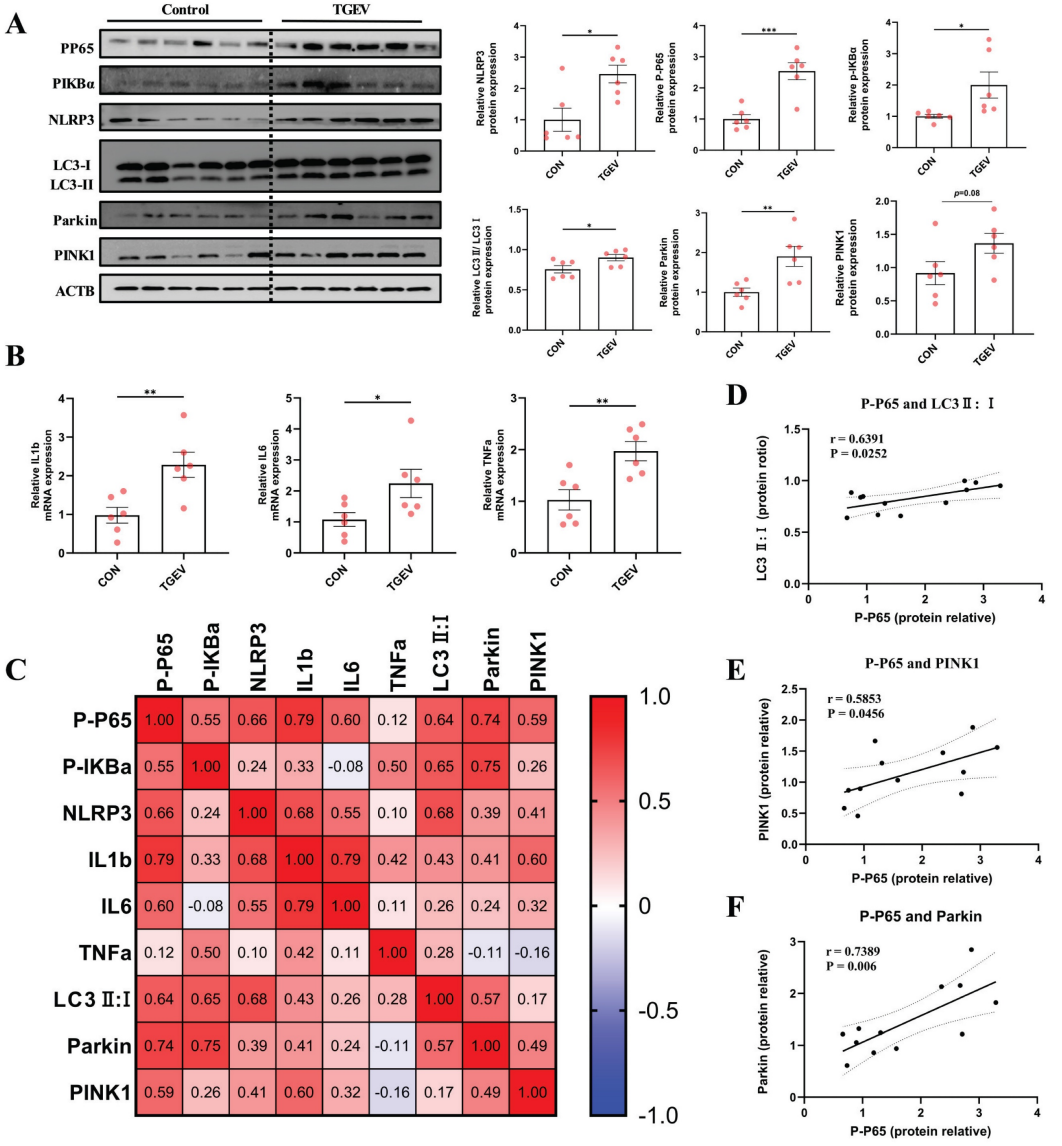
** TGEV-induced mitophagy is closely associated with the activation of inflammatory signaling.** Intestinal tissues were collected from TGEV-infected piglets 72 h post-infection for assessment of mitophagy markers and inflammatory signaling. (A) Western blot analysis of PINK1, Parkin, LC3, phosphorylated NF-κB p65, phosphorylated IκBα, and NLRP3 in jejunal tissues from control and TGEV-infected piglets (n = 6). (B) Quantitative RT-PCR analysis of *IL-1β*, *IL-6*, and *TNF-α* mRNA levels in intestinal tissues (n = 6). (C) Correlation heatmap showing Pearson correlation coefficients among P-p65, P-IκBα, NLRP3,* IL-1β*, *IL-6*, *TNF-α*, LC3-II/I ratio, Parkin, and PINK1 in jejunal tissues of piglets. (D-F) Pearson correlation scatter plots between P-p65 protein levels and LC3-II/I ratio (D), PINK1 (E), and Parkin (F). Data are presented as mean ± SEM. Correlation coefficients (r) and significance were calculated using Pearson's method.^ *^p < 0.05, ^**^p < 0.01, ^***^p < 0.001.

Western blot analysis revealed that TGEV infection significantly elevated the protein levels of PINK1 and Parkin, alongside an increased LC3-II/LC3-I ratio, indicating robust mitophagy activation *in vivo* (Figure 7A). These changes were accompanied by marked increases in P-P65, P-IκBα, and NLRP3 (*p* < 0.05), suggesting simultaneous upregulation of inflammatory pathways. Consistent with this, mRNA levels of *IL-1β*, *IL-6*, and *TNF-α* were significantly upregulated (*p* < 0.05) (Figure 7B), further confirming inflammatory activation.

To explore potential links between mitophagy and inflammation, we conducted correlation analyses. P-P65 levels positively correlated with LC3-II/I, PINK1, and Parkin expression, as well as with proinflammatory cytokine transcripts (Figure 7C-F). These *in vivo* findings parallel our *in vitro* data, supporting a model in which TGEV-induced mitophagy contributes to sustained intestinal inflammation, suggesting that targeting aberrant mitophagy may represent an effective strategy to mitigate TGEV-induced inflammatory injury.

### Quercetin protects against TGEV-induced intestinal inflammation by suppressing mitophagy-driven mitochondrial dysfunction *in vivo*

**Figure 8:**
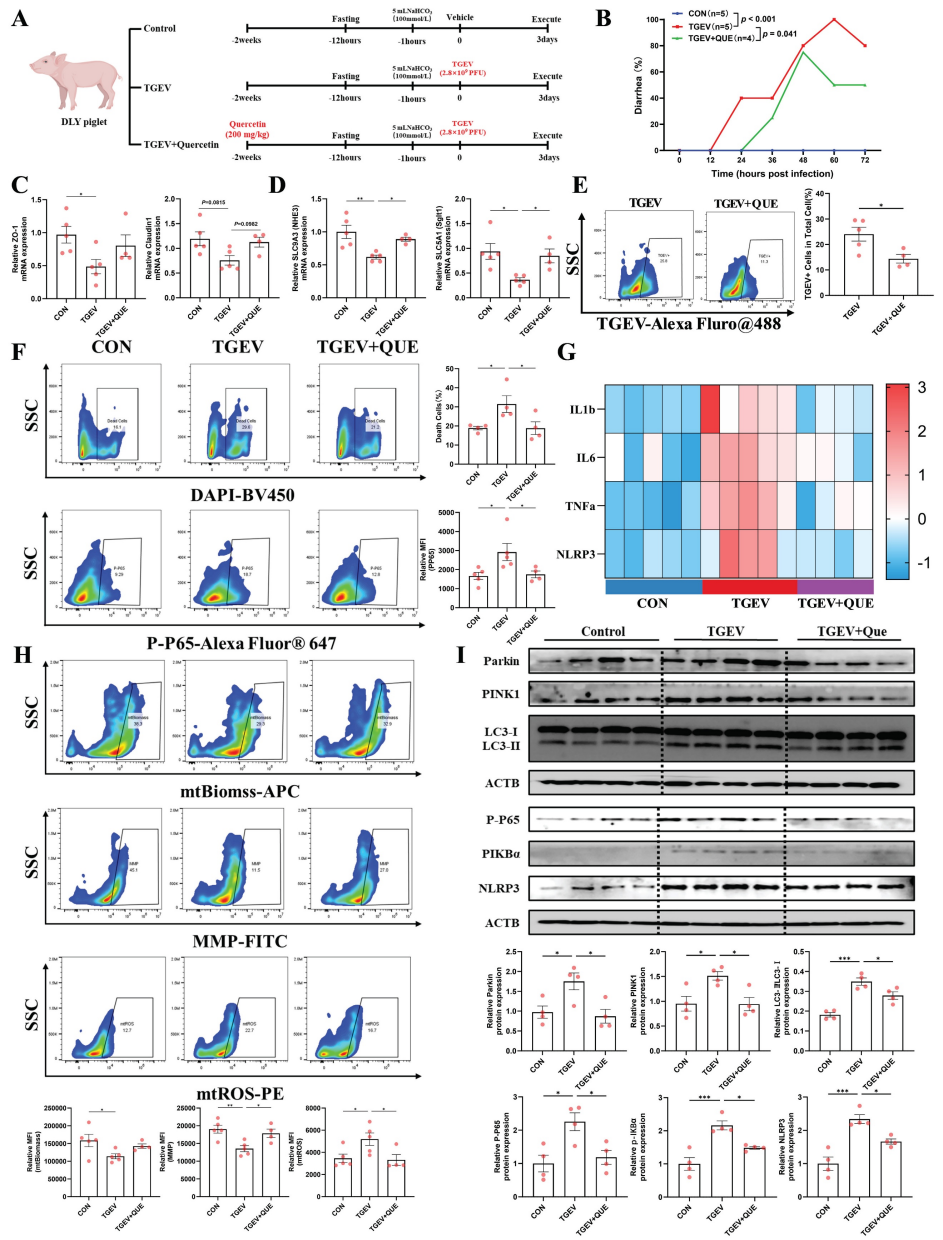
** Quercetin protects against TGEV-induced intestinal inflammation by suppressing mitophagy-driven mitochondrial dysfunction *in vivo*.** (A) Experimental design schematic illustrating the piglet model with dietary quercetin supplementation and TGEV oral challenge. (B) Diarrhea incidence rate of piglets from control, TGEV-infected, and TGEV+Quercetin groups. (C) mRNA expression of tight junction proteins *ZO-1* and *Claudin-1* in jejunal mucosa. (D) mRNA expression of diarrhea-associated electrolyte and nutrient transporters *SLC9A3* (*Nhe3*) and* SLC5A1* (*Sglt1*) in jejunal mucosa. (E) Flow cytometric analysis of TGEV infection in jejunal epithelial cells using an anti-TGEV nucleocapsid antibody labeled with Alexa Fluor 488. (F) Flow cytometric quantification of DAPI-positive dead cells and p-P65-positive inflammatory epithelial cells in jejunal epithelial cells. (G) mRNA expression levels of pro-inflammatory cytokines (*IL-1β*, *IL-6*, *TNF-α*, and *NLRP3*) visualized as a heatmap. (H) Flow cytometric analysis of mitochondrial function markers, including mtROS, MMP, and mtBiomass. (I) Western blot analysis and quantitative densitometry of proteins involved in mitophagy (PINK1, Parkin, LC3-II/I) and inflammation (P-P65, P-IκBα, NLRP3). Data were expressed as mean ±SEM. ^*^p < 0.05, ^**^p < 0.01, ^***^p < 0.001.

To validate the *in vivo* relevance of our findings, we established a piglet model orally challenged with TGEV, with or without dietary quercetin supplementation (Figure 8A). TGEV-infected piglets exhibited a significantly increased incidence of diarrhea (*p* < 0.001), while quercetin treatment effectively reduced the diarrhea rate (*p* < 0.05) (Figure 8B). Given the importance of intestinal barrier integrity in viral enteritis, we assessed the expression of tight junction proteins. TGEV infection led to a significant reduction in *ZO-1* mRNA (*p* < 0.05) and a decreasing trend in *Claudin-1* (*p* = 0.0815), indicating epithelial barrier disruption. Quercetin partially alleviated the reduction in *Claudin-1* (*p* = 0.0982) but did not significantly restore *ZO-1* expression (Figure 8C). We then investigated diarrhea-associated markers SLC9A3 (Nhe3) and SLC5A1 (Sglt1), two essential transporters involved in sodium-dependent electrolyte and nutrient absorption, critical for intestinal fluid homeostasis. Both markers were significantly downregulated following TGEV infection (*p* < 0.05), reflecting impaired intestinal absorptive function. Importantly, quercetin supplementation effectively restored their expression levels (*p* < 0.05), suggesting improved intestinal function (Figure 8D). By contrast, the mRNA levels of *Slc4a4* (*Nbce1*) and *Slc12a2* (*Nkcc1*), two basolateral ion transporters involved in bicarbonate uptake and chloride-driven electrolyte transport, respectively, remained largely unchanged among groups (Figure S3). Consistently, flow cytometry using an anti-TGEV-N antibody revealed a reduced percentage of TGEV-positive epithelial cells in the quercetin group (*p* < 0.05), indicating reduced viral load (Figure 8E).

To further explore the protective mechanisms of quercetin, we evaluated epithelial cell viability, inflammatory responses, and mitochondrial function. TGEV infection increased the proportions of DAPI-positive dead cells and p-P65-positive inflammatory epithelial cells (*p* < 0.05), both significantly decreased by quercetin supplementation (*p* < 0.05) (Figure 8F). mRNA expression analysis revealed that TGEV upregulated pro-inflammatory cytokines *IL-1β*, *IL-6, TNF-α*, and* NLRP3*, while quercetin effectively suppressed their expression (Figure 8G). Meanwhile, TGEV infection induced mitochondrial dysfunction, characterized by increased mitochondrial ROS, reduced membrane potential, and decreased mitochondrial biomass (*p* < 0.05). Quercetin significantly alleviated these impairments, preserving mitochondrial integrity (*p* < 0.05) (Figure 8H). Western blot analysis further showed that TGEV activated PINK1/Parkin-mediated mitophagy, as indicated by increased levels of PINK1, Parkin, and an elevated LC3-II/I ratio, accompanied by enhanced NF-κB and NLRP3 signaling (*p* < 0.05). Quercetin treatment reversed these changes, suppressing mitophagy overactivation and downstream inflammatory responses (*p* < 0.05) (Figure 8I). Collectively, these findings demonstrate that quercetin protects against TGEV-induced intestinal injury by mitigating mitophagy-driven mitochondrial dysfunction, thereby preserving intestinal function and reducing inflammation.

## Discussion

Coronaviruses have emerged as major threats to both human and animal health, with TGEV, a porcine α-coronavirus, serving as a useful model for studying intestinal coronavirus infections 4,6. TGEV is transmitted via the fecal-oral route and infects intestinal epithelial cells, where it replicates extensively, leading to severe epithelial damage—a pathogenic mechanism common across several coronaviruses 35. In addition to direct viral cytopathic effects, immune-mediated damage driven by excessive inflammatory responses also contributes critically to coronavirus pathogenesis. Against this background, plant-derived compounds have gained significant attention in antiviral research due to their broad-spectrum activity, safety, and multi-target mechanisms 36,37. Here, we identified quercetin from 20 plant extracts as a potent inhibitor of inflammation and mitochondrial damage during TGEV infection. Mechanistically, quercetin was found to suppress excessive mitophagy through inhibition of the PINK1/Parkin pathway, thereby preserving mitochondrial integrity and mitigating inflammation in TGEV-infected intestinal cells. These findings highlight the role of mitochondrial quality control in viral pathogenesis and underscore quercetin's therapeutic potential as a modulator of virus-induced cellular damage.

TGEV primarily targets intestinal epithelial cells, resulting in severe structural damage and functional dysregulation of the intestinal barrier 38. The virus-induced inflammatory response and cytopathic effects (CPEs) are considered central to its pathogenic process 39-41. Consistent with previous reports 42, our findings demonstrated significant TGEV replication in porcine intestinal epithelial cells (IPEC-J2), leading to extensive cellular damage and decreased cell viability. Our systematic screening of plant-derived compounds revealed quercetin as notably effective in counteracting TGEV infection. Beyond merely improving cell viability and reducing structural damage, quercetin substantially decreased viral load, suggesting a potent direct or indirect antiviral mechanism. While quercetin has previously been reported to exert antiviral effects against influenza virus 43, Zika virus 44, and SARS-CoV-2 45, its role in TGEV infection has remained unexplored. Our findings extend its antiviral spectrum and establish quercetin as a potent inhibitor of TGEV replication in porcine IECs.

Beyond direct viral neutralization, many plant-derived compounds are known to confer antiviral effects through host-directed mechanisms, including attenuation of inflammatory responses, regulation of oxidative stress, and modulation of cellular stress signaling 46,47. Our data suggest that quercetin does not impair TGEV infectivity per se but acts through pre-conditioning the host epithelial environment to resist infection. This implies that enhancing host resilience, rather than neutralizing the virus directly, may be a viable strategy in managing intestinal coronavirus infections. Publicly available transcriptomic data and network pharmacology analysis revealed that quercetin may modulate TGEV infection by targeting overlapping genes enriched in key inflammatory pathways, including NF-κB, JAK/STAT, and TNF-α. These pathways are consistently activated in response to coronavirus infections and play pivotal roles in orchestrating cytokine release and immune cell recruitment 48. Given the well-established role of these pathways in amplifying pro-inflammatory cascades, their modulation by quercetin likely contributes to the overall attenuation of the inflammatory milieu. This reduction in inflammation, in turn, alleviates epithelial injury during viral infection. Moreover, the localization of key targets within mitochondrial and nuclear compartments provides mechanistic context to our functional data, in which quercetin preserved mitochondrial homeostasis while dampening inflammatory activation. Mitochondria are increasingly recognized as central immunometabolic hubs 49,50. Thus, therapeutic strategies that preserve mitochondrial integrity while simultaneously suppressing pro-inflammatory transcriptional programs may provide dual protective benefits against viral infections. Mitochondrial dysfunction is a prominent and early event in TGEV pathogenesis, occurring prior to overt inflammatory activation. In our study, mitochondrial impairment was evident as early as 3 hours post-infection, as indicated by a significant decline in mitochondrial biomass. This preceded the transcriptional upregulation of inflammatory cytokines, including *IL-1β*, *IL-6*, *IL-18*, and *TNF-α*, which only became prominent at 12-24 hours post-infection. These cytokines are key mediators of TGEV-induced intestinal injury, as they amplify mucosal inflammation, compromise epithelial barrier integrity, and promote enterocyte apoptosis. The temporal disconnect between mitochondrial collapse and cytokine induction suggests that mitochondrial damage may act as an upstream trigger of the inflammatory cascade, rather than a consequence of it. This interpretation is further supported by concurrent evidence of mitochondrial depolarization, loss of mtDNA content, and accumulation of mtROS—hallmarks of mitochondrial stress known to activate innate immune pathways 51-54. These mitochondrial abnormalities were accompanied by activation of NF-κB and JAK-STAT signaling pathways, which in turn were associated with increased expression of pro-inflammatory genes such as *TNF-α*, *IL-6*, *IL-1β*, and *NLRP3*. Importantly, treatment with quercetin reversed these early mitochondrial defects and suppressed the subsequent inflammatory amplification, pointing toward a causative role of mitochondrial dysfunction in driving TGEV-induced epithelial inflammation. Taken together, these findings suggest that mitochondrial instability is not merely associated with TGEV-induced inflammation but may represent an initiating event in the pathogenic sequence.

Given the early onset of mitochondrial dysfunction during TGEV infection, we sought to determine whether the anti-inflammatory effects of quercetin are mechanistically dependent on its ability to preserve mitochondrial integrity. Functional interference experiments supported this notion. Co-treatment with the mitochondrial antioxidant NAC enhanced quercetin's anti-inflammatory effects, while rotenone-induced mitochondrial dysfunction abolished them. This dependency suggests that mitochondrial integrity acts as an upstream modulator of inflammation during TGEV infection. Mitochondria, beyond their metabolic role, serve as immunological signaling hubs, where redox imbalance—particularly mitochondrial ROS overproduction—activates NLRP3 inflammasomes and NF-κB signaling cascades 55. In this context, preserving mitochondrial homeostasis is not merely beneficial for cellular survival, but also critical in preventing pathological immune activation 56,57. Consistent with this framework, quercetin restored key mitochondrial parameters, including membrane potential, mtDNA stability, and expression of respiratory chain components. These mitochondrial-stabilizing effects likely underlie its capacity to attenuate inflammatory responses, by reducing ROS accumulation and limiting downstream inflammasome activation. Previous studies further support this link, showing that mitochondrial destabilization amplifies pro-inflammatory cytokine cascades and aberrant immune signaling 58,59.

The identification of mitophagy as a proximal driver of mitochondrial collapse and downstream inflammation in TGEV infection adds an important dimension to our understanding of viral immunopathology. While basal mitophagy is essential for mitochondrial turnover and cytoprotection 60, excessive activation can paradoxically accelerate mitochondrial depletion and trigger persistent inflammatory signaling 61,62. Our data support this dichotomy, showing that TGEV robustly activates PINK1/Parkin-mediated mitophagy, leading to mtDNA loss, impaired electron transport chain activity, and heightened ROS accumulation—all of which contribute to sustained activation of NF-κB and NLRP3 pathways. What distinguishes our study from prior work is the temporal and functional positioning of mitophagy upstream of mitochondrial dysfunction and inflammation. Through pharmacological modulation using CCCP and Mdivi-1, we demonstrate that quercetin's anti-inflammatory effects are at least partially dependent on suppression of aberrant mitophagic flux. When mitophagy was re-induced by CCCP, quercetin lost its ability to inhibit NF-κB and NLRP3 activation. Conversely, mitophagy inhibition by Mdivi-1 enhanced quercetin's protective effects, indicating that mitophagy is not merely a parallel response to stress but a causal mediator of TGEV-induced pathogenesis. Importantly, the pathological role of mitophagy in this context differs from its traditionally protective function. This contradiction likely reflects a tipping point in the mitophagic response—a threshold beyond which quality control shifts into maladaptive self-destruction. Such a shift has been observed in chronic inflammatory and metabolic diseases 63,64, but its implication in intestinal coronavirus infection has not been well defined. Our findings therefore not only elucidate a novel mitophagy-inflammation axis in TGEV pathology, but also identify quercetin as a pharmacological tool capable of restoring this axis to a homeostatic setpoint. Rather than merely responding to downstream inflammatory signals, quercetin interrupts an earlier, mitophagy-initiated pathological loop at the level of mitochondrial quality control. This upstream targeting strategy holds broader relevance beyond TGEV, as other coronaviruses including SARS-CoV-2 have also been shown to manipulate autophagic processes to evade immune detection 65. The concept of "mitophagy modulation" as a therapeutic checkpoint thus warrants further investigation in diverse viral and inflammatory models.

To determine whether the mitophagy-mitochondrial stress-inflammation axis observed *in vitro* also operates under physiological conditions, we evaluated intestinal tissues from TGEV-infected weaned piglets. This model is of particular relevance, as TGEV-induced enteritis is characterized by rapid epithelial destruction and acute mucosal inflammation in young animals 12,66, and provides a more integrated view of viral pathogenesis within a multicellular environment. Our observations suggest that PINK1/Parkin-mediated mitophagy and downstream inflammatory responses are co-activated in TGEV-infected intestinal tissues, supporting the concept that excessive mitophagy may contribute to epithelial damage not merely as a bystander, but as a driver of immune dysregulation. The strong correlation between mitophagy and inflammatory markers suggest that excessive mitophagy is not an artifact of *in vitro* stress conditions but a pathophysiological process that may contribute meaningfully to tissue-level immune dysregulation during intestinal coronavirus infection. This *in vivo* evidence not only strengthens the credibility of our hypothesis—proposing that mitochondrial dysfunction promotes inflammation—but also underscores the potential pathogenic role of PINK1/Parkin-mediated mitophagy in TGEV-associated intestinal injury.

In a follow-up *in vivo* experiment incorporating dietary quercetin supplementation, we further validated its protective effects against TGEV-induced intestinal injury in piglets. Beyond reducing diarrhea incidence and viral burden, quercetin effectively restored epithelial absorptive function—evidenced by the rescue of *SGLT1* and *NHE3*—and partially preserved tight junction integrity, highlighting its role in maintaining both structural and functional components of the intestinal barrier. This is particularly relevant, as the hallmark symptom of TGEV infection—profuse watery diarrhea—stems primarily from disrupted epithelial electrolyte and nutrient transport 67,68. Beyond phenotypic improvements, our data provide evidence for a mechanistic link whereby quercetin disrupts a feed-forward loop of epithelial injury, mitochondrial dysfunction, and inflammatory amplification. By suppressing excessive PINK1/Parkin-mediated mitophagy, quercetin preserved mitochondrial function and attenuated downstream activation of NF-κB and NLRP3 signaling—thereby halting the amplification of inflammatory damage at its source. This mechanism aligns with emerging evidence that excessive mitophagy, while initially protective, can become maladaptive under sustained stress 69. These findings underscore the significance of modulating PINK1/Parkin-dependent mitophagy as a therapeutic strategy in coronavirus-induced intestinal disease, highlighting quercetin's capacity to restore epithelial homeostasis by stabilizing mitochondrial function and limiting inflammation.

In summary, our study systematically elucidates the pathogenic mechanism of TGEV-induced intestinal inflammation from the perspective of mitochondrial quality control. We demonstrate that TGEV infection activates the PINK1/Parkin-mediated mitophagy pathway both *in vitro* and *in vivo*, leading to mitochondrial dysfunction, NF-κB signaling activation, and proinflammatory cytokine release. Importantly, we identify quercetin, screened from a panel of 20 plant-derived compounds, as a potent antiviral candidate that alleviates TGEV-induced inflammation by restoring mitochondrial function and suppressing excessive mitophagy. Functional interference assays using NAC, rotenone, CCCP, and Mdivi-1 further confirm that the anti-inflammatory effects of quercetin are dependent on its ability to maintain mitochondrial homeostasis and inhibit aberrant mitophagic flux. Collectively, these findings highlight a previously unrecognized role of mitophagy in mediating coronavirus-induced intestinal injury and provide compelling evidence that targeting mitochondrial dynamics—particularly via natural plant compounds like quercetin—may offer promising therapeutic avenues against TGEV and potentially other enteropathogenic coronaviruses.

## Supplementary Material

Supplementary figures and tables.
