# Supplementary material for: Quercetin Protects Against Transmissible Gastroenteritis Virus-Induced Intestinal Inflammation by Modulating Mitophagy-Driven Mitochondrial Dysfunction
Source: Int J Biol Sci. 2025 Oct 10;21(15):6559–78. doi: 10.7150/ijbs.116855 (PMC12631068; doi:10.7150/ijbs.116855)
Supplement: Supplementary file 1 — Supplementary figures and tables. [file ijbsv21p6559s1.pdf]

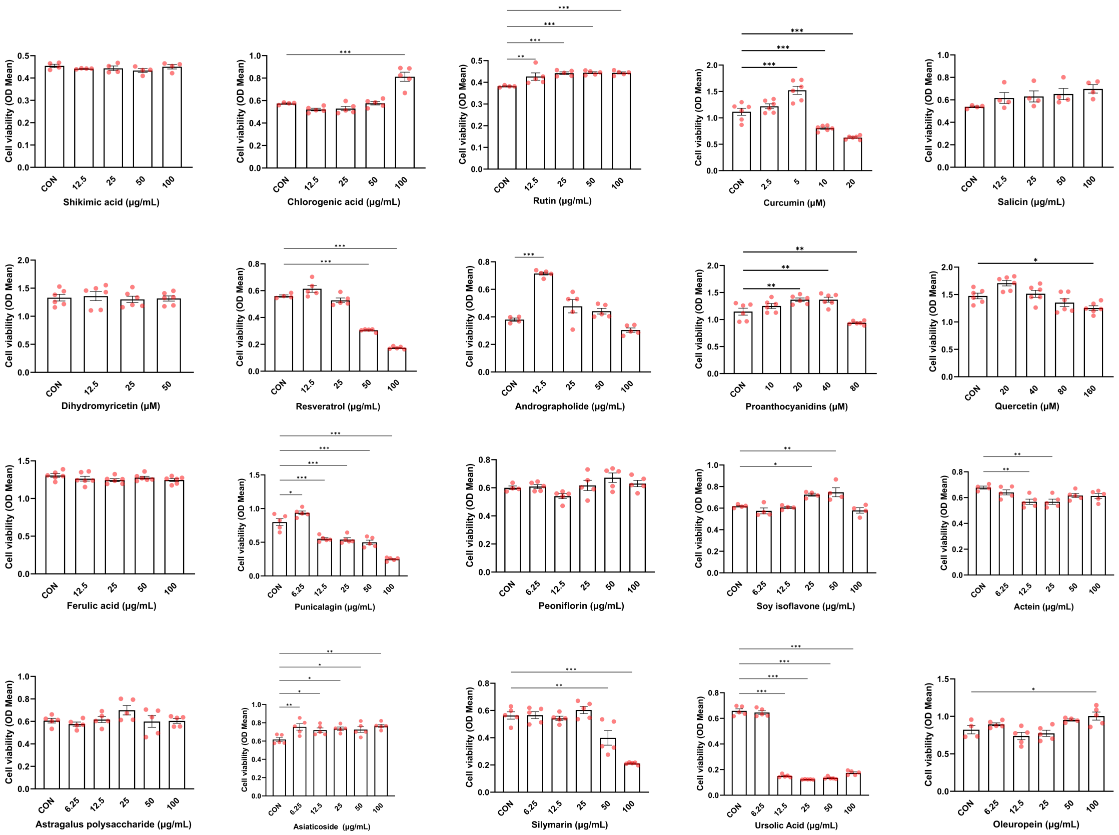

Figure S1 Cytotoxicity assessment of 20 plant-derived compounds in IPEC-J2 cells. Cell viability was evaluated using the CCK-8 assay following 24 h treatment with increasing concentrations of each compound. Data are presented as mean ± SEM (n = 4-6). $^{*}p < 0.05$, $^{**}p < 0.01$, $^{***}p < 0.001$.

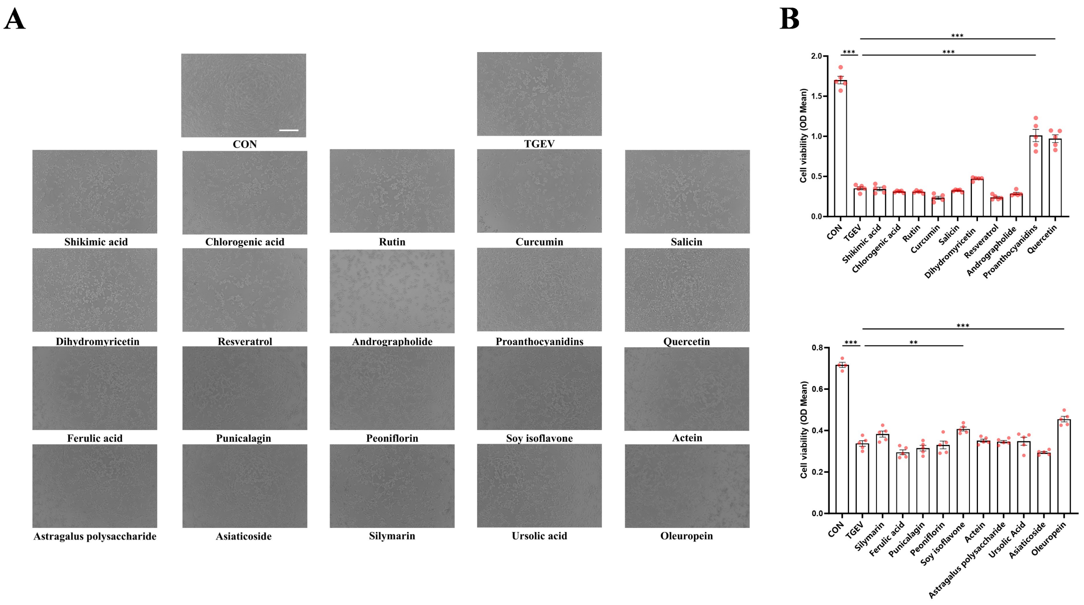

Figure S2 Screening and evaluation of natural compounds for antiviral effects against TGEV in IPEC-J2 cells (A) Representative phase-contrast images showing cell morphology in control (CON), TGEV-infected, and compound-treated groups. Scale bar, 100 μm. (B) CCK-8 assay evaluating cell viability. (n = 5-6). The working concentrations of the tested compounds were: shikimic acid (100 μg/mL), chlorogenic acid (100 μg/mL), rutin (100 μg/mL), curcumin (5 μM), salicin (100 μg/mL), dihydromyricetin (50 μM), resveratrol (25 μg/mL), andrographolide (100 μg/mL), proanthocyanidins (40 μM), quercetin (80 μM), ferulic acid (100 μg/mL), punicalagin (6.25 μg/mL), peoniflorin (100 μg/mL), soy isoflavone (100 μg/mL), actein (100 μg/mL), astragalus polysaccharide (100 μg/mL), asiaticoside (100 μg/mL), silymarin (25 μg/mL), ursolic acid (6.25 μg/mL), and oleuropein (100 μg/mL). Data are presented as mean ± SEM (n = 5-6). $^{*}p < 0.05$, $^{**}p < 0.01$, $^{***}p < 0.001$.

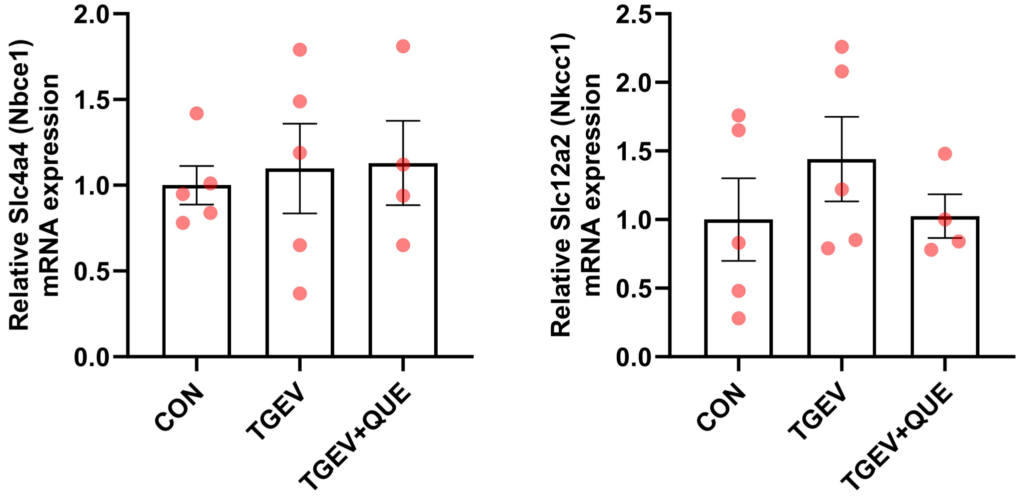

Figure S3 mRNA expression of diarrhea-associated electrolyte and nutrient transporters *Slc4a4* (*Nbce1*) and *Slc12a2* (*Nkcc1*) in jejunal mucosa. Data are presented as mean ± SEM.

Table S1 List of Chemicals and Antibodies

REAGENT or RESOURCE	SOURCE	IDENTIFIER
**Chemicals**		
Resveratrol	Layn Bio	CAS:501-36-0
Dihydromyricetin	Layn Bio	CAS:27200-12-0
Chlorogenic acid	Layn Bio	CAS:327-97-9
Ferulic acid	Layn Bio	CAS:537-98-4
Quercetin	Layn Bio	CAS:117-39-5
Proanthocyanidins	Layn Bio	CAS:222838-60-0
Curcumin	Layn Bio	CAS:458-37-7
Andrographolide	Layn Bio	CAS:5508-58-7
Rutin	Layn Bio	CAS:153-18-4
Shikimic acid	Layn Bio	CAS:138-59-0
Salicin	Layn Bio	CAS:138-52-3
Punicalagin	Layn Bio	CAS:65995-63-3
Peoniflorin	Layn Bio	CAS:23180-57-6
Soy isoflavone	Layn Bio	N/A
Asiaticoside	Layn Bio	CAS:16830-15-2
Silymarin	Layn Bio	CAS:65666-07-1
Ursolic acid	Layn Bio	CAS:77-52-1
Oleuropein	Layn Bio	CAS:32619-42-4
Astragalus polysaccharide	Layn Bio	N/A
Actein	Layn Bio	CAS:18642-44-9
Acetylcysteine (NAC)	MedChemExpress	Cat#HY-B0215
Rotenone	TargetMol	Cat#T2970
CCCP	TargetMol	Cat#T7081
Mdivi-1	TargetMol	Cat#T1907
MitoSOX Red	MedChemExpress	Cat#HY-D1055
Rhodamine 123	MedChemExpress	Cat#HY-D0816
Lyso-Tracker Green	Beyotime	Cat#C1047
Mito-Tracker Deep Red FM	Beyotime	Cat#C1032
DAPI	Beyotime	Cat#P0131
**Antibodies**		
NF-κB p65 XP® Rabbit mAb	CST	Cat#8242
Phospho-NF-κB p65 (Ser536)Rabbit mAb	CST	Cat#3033
IκBα Antibody	CST	Cat#9242
IκB-α (Phospho Ser32/36) Rabbit pAb	Immunoway	Cat#YP0151
NLRP3 Rabbit mAb	ABclonal	Cat#A24294
PINK1 Rabbit mAb	Selleck	Cat#F0490
Parkin Rabbit mAb	Selleck	Cat#F0296
Anti-SQSTM1 / p62 antibody	Abcam	Cat#ab56416
Beclin-1 Antibody	CST	Cat#3738
Stat1 (D1K9Y) Rabbit mAb	CST	Cat#14994

Phospho-Stat1 (Tyr701) (D4A7) Rabbit mAb	CST	Cat#7649
Stat3 (124H6) Mouse mAb	CST	Cat#9139
Phospho-Stat3 (Tyr705) (D3A7) XP® Rabbit mAb	CST	Cat#9145
HO-1/HMOX1 Polyclonal antibody	Proteintech	Cat#10701-1-AP
Complex I (NDUFA9) Antibody	GeneTex	Cat#GTX132978
Complex II (SDHA) Antibody	GeneTex	Cat#GTX636098
Complex III (UQCRC1) Antibody	GeneTex	Cat#GTX630393
Complex IV (COX5A) Antibody	Servicebio	Cat#GB111676
Complex V (ATP5B) Antibody	GeneTex	Cat#GTX132925
β-Actin (8H10D10) Mouse mAb	CST	Cat#3700
TGE Virus Mouse mAb	Santa Cruz	Cat#sc-52436

Table S2. Primer sequences table.

Gene	Primers	Sequences	Product size	Accession numbers
β-actin	Forward	GCAAATGCTTCTAGGCGGAC	148	XM_021086047.1
	Reverse	GCGTCCATCACAGCTTCTCA		
NLRP3	Forward	CTTCTTGAAAACTATGGCAAA	102	NM_001256770.2
	Reverse	TTTCTCCAAGTAGGACGTT		
IL-1β	Forward	AGCCAGTCTTCATTGTTCAGGT	132	NM_214055.1
	Reverse	TCATCTCTTTGGGGCCATCAG		
IL-18	Forward	AGCTGAAAACGATGAAGACCTG	121	NM_213997.1
	Reverse	AAACACGGCTTGATGTCCCT		
mtDNA	Forward	GCCTTGCCAAACCCCAAAAA	137	AF276923.1
	Reverse	TAGGTGCCTGCTTTCGTAGC		
IL-6	Forward	TTCAGTCCAGTCGCCTTCTCC	97	NM_214399.1
	Reverse	TGGCATCACCTTTGGCATCTTC		
IFNB1	Forward	GAGCTATGATGTGCTTCGAT	139	NM_001003923.1
	Reverse	TGGTTGCATAATCTCCTCAG		
CXCL10	Forward	CATGTTGAGATCATTGCCAC	202	NM_001008691.1
	Reverse	TGTAGAGAGATGATGGCAGA		
TNFa	Forward	CATCGTCTCAAACCTCAGAT	172	NM_214022.1

	Reverse	TGGGAGTAGATGAGGTACAG		
TGEV	Forward	ACGCTTGGTAGTCGTGGTG	171	JX827607.1
	Reverse	GGATTGTTGCCTGCCTCT		
SLC9A3	Forward	CCTCACGCTGTGGACACTC	194	XM_021077062.1
	Reverse	GGGGCAGCAGGTAGAAGAAG		
SLC5A1	Forward	CGTCATCTACTTCGTGGTGGT	104	NM_001164021.1
	Reverse	ACACCATACTTCGTCCAGCC		
Slc12a2	Forward	ATCGCCTGGTGTCAAGGATG	135	XM_003123899.5
	Reverse	GGTTGAGTTGGAGTCTTGCC		
Slc4a4	Forward	CACCGGCATCAAACCAAGAA	105	NM_001030533.1
	Reverse	AGGGCTACCATTCTCAGGGC		
ZO1	Forward	CAGCCCCCGTACATGGAGA	114	XM_021098896.1
	Reverse	GCGCAGACGGTGTTCATAGTT		
Claudin1	Forward	TCTTAGTTGCCACAGCATGG	106	NM_001244539.1
	Reverse	CCAGTGAAGAGAGCCTGACC		
